# Fluorescence-Based Methods for Detecting Caries Lesions: Systematic Review, Meta-Analysis and Sources of Heterogeneity

**DOI:** 10.1371/journal.pone.0060421

**Published:** 2013-04-04

**Authors:** Thais Gimenez, Mariana Minatel Braga, Daniela Procida Raggio, Chris Deery, David N. Ricketts, Fausto Medeiros Mendes

**Affiliations:** 1 Department of Pediatric Dentistry, School of Dentistry, University of São Paulo-USP, São Paulo, Brazil; 2 School of Clinical Dentistry, University of Sheffield, Sheffield, United Kingdom; 3 Dundee Dental Hospital and School, University of Dundee, Dundee, United Kingdom; University of Toronto, Canada

## Abstract

**Background:**

Fluorescence-based methods have been proposed to aid caries lesion detection. Summarizing and analysing findings of studies about fluorescence-based methods could clarify their real benefits.

**Objective:**

We aimed to perform a comprehensive systematic review and meta-analysis to evaluate the accuracy of fluorescence-based methods in detecting caries lesions.

**Data Source:**

Two independent reviewers searched PubMed, Embase and Scopus through June 2012 to identify papers/articles published. Other sources were checked to identify non-published literature.

**Study Eligibility Criteria, Participants and Diagnostic Methods:**

The eligibility criteria were studies that: (1) have assessed the accuracy of fluorescence-based methods of detecting caries lesions on occlusal, approximal or smooth surfaces, in both primary or permanent human teeth, in the laboratory or clinical setting; (2) have used a reference standard; and (3) have reported sufficient data relating to the sample size and the accuracy of methods.

**Study Appraisal and Synthesis Methods:**

A diagnostic 2×2 table was extracted from included studies to calculate the pooled sensitivity, specificity and overall accuracy parameters (Diagnostic Odds Ratio and Summary Receiver-Operating curve). The analyses were performed separately for each method and different characteristics of the studies. The quality of the studies and heterogeneity were also evaluated.

**Results:**

Seventy five studies met the inclusion criteria from the 434 articles initially identified. The search of the grey or non-published literature did not identify any further studies. In general, the analysis demonstrated that the fluorescence-based method tend to have similar accuracy for all types of teeth, dental surfaces or settings. There was a trend of better performance of fluorescence methods in detecting more advanced caries lesions. We also observed moderate to high heterogeneity and evidenced publication bias.

**Conclusions:**

Fluorescence-based devices have similar overall performance; however, better accuracy in detecting more advanced caries lesions has been observed.

## Introduction

The prevalence of dental caries (tooth decay) and its progression have decreased in recent years [1], [2]. With this background in mind, the early diagnosis of caries is thought to be difficult and the changes in the presentation of the disease may be making diagnosis worse [3]. Visual inspection (clinical examination) is the method of choice in daily clinical practice for detecting caries lesions [4], [5]. However, despite the high specificity (correct identification of sound sites), visual inspection has achieved sub-optimal sensitivity (correct identification of carious sites) and reproducibility values [6]. As a result, adjunct methods of caries detection have been proposed to improve the accuracy and reproducibility of caries detection and in some cases to allow for more objective assessment.

The most common adjunct method for caries detection in clinical practice is radiography, however, more recently several fluorescence-based methods have been used to aid and inform the caries detection and diagnostic process. These methods are based on the principle that carious dental tissues have altered (decreased) fluorescence properties compared with sound dental tissues. The quantitative light-induced fluorescence method of caries detection (QLF, Inspektor, Amsterdam, The Netherlands) uses a halogen lamp which emits a blue light with a wavelength of 370 nm that excites the tooth structure which then fluoresces. The fluorescent images are then captured and software quantifies the loss of fluorescence provoked by the demineralization within carious lesions. Reduction in the fluorescence indicates mineral loss [7].

Another laser fluorescence (LF) method is based on the emission of a red light, with a wavelength of 655 nm, through a diode laser. The light reaches the dental tissues, which emits fluorescence in the near-infrared range. The first device that was made commercially available utilising this technique captures the fluorescence and translates its intensity into a relative numerical scale from 0 to 99 [8]. This device was introduced onto the market to detect occlusal and smooth-surface caries lesions (DIAGNOdent, Kavo, Biberach, Germany) however, this was superseded by a cable free pen-type laser fluorescence device (LFpen) which additionally allowed approximal surfaces to be examined (DIAGNOdent pen, Kavo, Biberach, Germany) [9], [10]. Both devices are based on the physical property that carious tissue fluoresces more strongly, mainly due to bacterial porphyrins, than sound tissue when excited by visible light at this wavelength [4], [9].

More recently, a fluorescence camera (FC; Vista Proof, Dürr Dental, Germany), has been developed for caries detection on occlusal surfaces. The tool emits a light with a 400-nm wavelength and filters the fluorescence emitted by the tissue. Specific software then quantifies the fluorescence on a numerical scale from 0 to 5. This device also captures the fluorescence from bacterial porphyrins [11].

Several studies have evaluated the performance of these methods in detecting and quantifying carious lesions. The range of reported results is extensive and contradictory. Systematic reviews are important to summarize the advances in health care for practitioners, in order to ensure the correct implementation and adoption of research knowledge in everyday practice for the benefit of patients. They also identify the areas where there are gaps in knowledge. Thus, the aim of this study therefore was to synthesize the findings about the accuracy of fluorescence-based methods in detecting caries lesions on occlusal, approximal and smooth surfaces of both permanent and primary teeth by conducting a comprehensive systematic review and meta-analysis. We also investigated possible sources of heterogeneity and publication bias. This is the first known systematic review of diagnostic methods of caries lesions that has performed a series of meta-analyses and meta-regressions to evaluate overall accuracy and possible reasons for heterogeneity.

## Materials and Methods

To conduct this review, we followed the guideline “Preferred reporting items for systematic reviews and meta-analyses (PRISMA)” [12]. The PRISMA checklist is included as Supporting Information (Table S2).

### Information sources

We performed the literature search in MEDLINE (PubMed) for articles published until 19^th^, June 2012 that reported accuracy in detecting caries lesions by one of the following fluorescence-based methods: QLF, LF, LFpen or FC. Similar searches were done using the Embase and Scopus databases. To reduce publication bias, unpublished documents were pursued through OpenSIGLE and the Annals of ORCA Congress (European Organisation for Caries Research) for the last 10 years. The references of the articles included were also checked for verification of possible items not identified by the search. No restrictions were made with respect to the study design.

### Search

**Figure 1:**
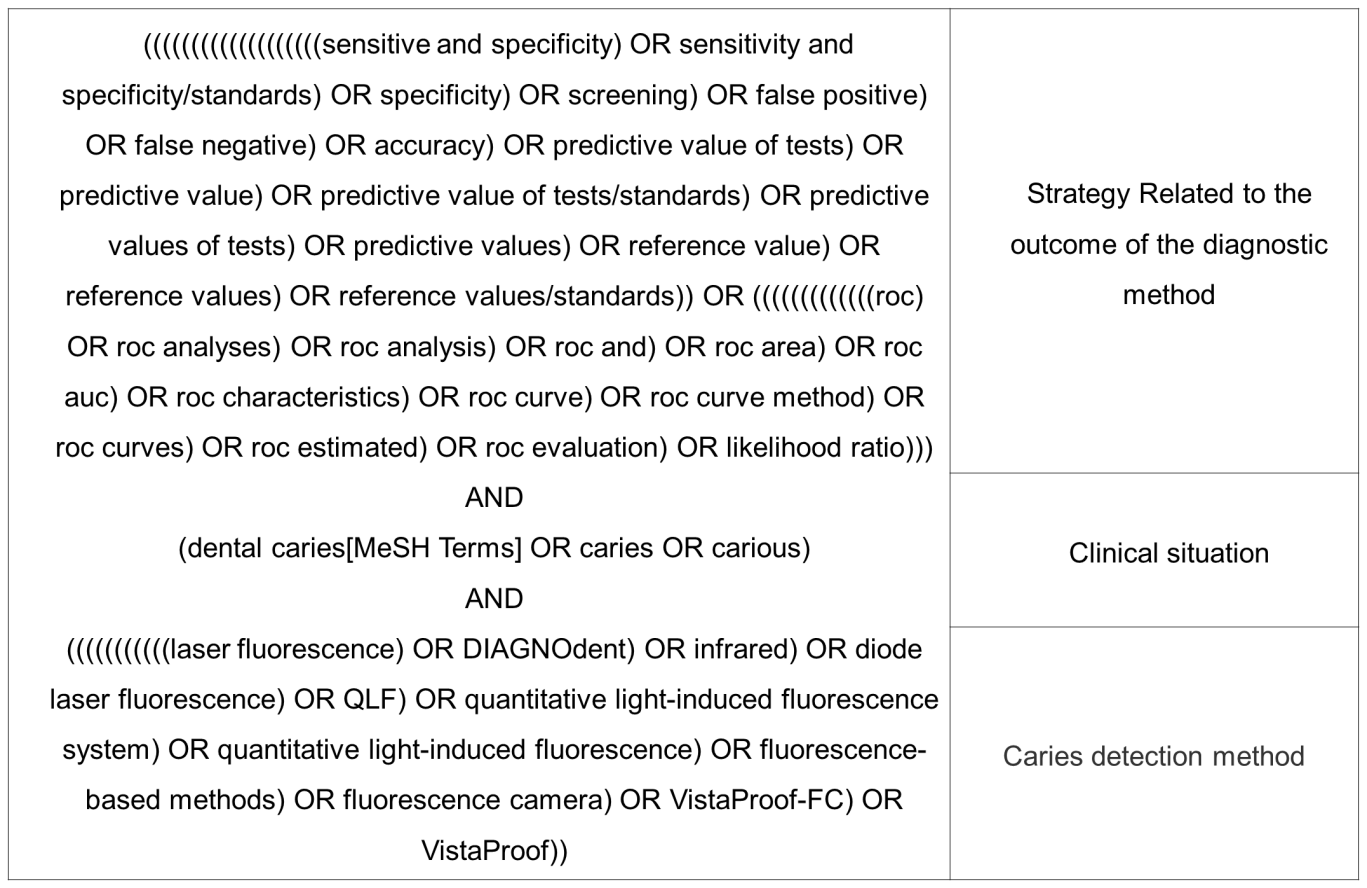
Search strategy. Chart containing the search strategy for electronic databases.

We divided the search of electronic databases into three parts, for illustrative purposes. The first part corresponded to the optimal search strategy for diagnostic studies [13]. The second was related to the clinical situation under investigation (caries lesions) and the third was associated with the caries detection method (Figure 1). Each part was associated to the other with the Boolean tool “AND”. The syntax was developed to search in the MEDLINE database and was adapted for other databases.

The results of searches of various databases were cross checked, in order to locate and eliminate duplicates.

### Study Selection and Eligibility criteria

After locating the studies, the titles and abstracts were examined to ensure they fulfilled the following inclusion criteria: (1) studies that mentioned some fluorescence-based methods (LF, LFpen, FC or QLF) in detecting primary caries lesions; and (2) studies ( that used human teeth, either in vitro or in vivo, primary or permanent teeth and on smooth, approximal or occlusal surfaces.

The articles whose titles and abstracts met these inclusion criteria were then searched to ensure there was a reference standard (gold standard) and they reported the absolute numbers of true positives (TP), false positives (FP), true negatives (TN) and false negatives (FN) or presented sufficient data to derive these figures.

Two reviewers (TG and MMB) independently identified potential references and eliminated irrelevant studies. Doubts or disagreements were solved by discussion with a third researcher (FMM). Studies that used the same data set for more than 1 publication were included only once in this review. Articles that reported diagnosis of root or artificially developed caries lesions, as well as, caries lesions around restorations, were excluded.

### Data collection process

Data were extracted by one reviewer (TG) directly from the full texts of articles to structured tables containing all variables and data about accuracy. A second researcher (FMM) independently verified the extracted data. Discrepancies were solved by checking the source and discussion. Whenever possible, we extracted raw data from primary studies to fill a diagnostic 2×2 table. When studies did not provide confidence intervals for sensitivity or specificity, we estimated them using Review Manager Software (RevMan Version 5.1, The Nordic Cochrane Centre, The Cochrane Collaboration, Copenhagen, Denmark).

The following information was extracted from papers: diagnostic method, reference standard test, cut-offs values, setting (in vivo or in vitro studies and in case of in vitro studies, if specimens had been stored frozen or not), type of teeth (primary or permanent), surface evaluated (smooth, approximal or occlusal), sample size and outcome data (sensitivity and specificity). In some articles, the values of TP, TN, FP and FN were available. If not, we derived the numbers from the sample size, caries prevalence and reported sensitivity and specificity. If a study reported pairs of sensitivities and specificities at different cut-off points, we extracted the pair with the highest values (optimal cut-off). If the study evaluated the performance of the method with more than one examiner, only the values of the first examiner were considered. Unfortunately, this can lead to loss of accuracy data. However, this strategy was adopted based on a medical systematic review aiming to prevent the duplication of sample data (cluster effect), which could lead to bias [14]. If the study reported the interference of different variables on the performance of the method, only baseline values were annotated.

### Risk of bias of individual studies

We used a modified QUADAS (Quality assessment of studies of diagnostic performance included in systematic reviews) checklist to assess the quality of included studies [15], but there was no intention to classify the studies. We only used these quality items to asses possible sources of heterogeneity [16]. This modified version consists of 11 items on methodological characteristics that have the potential to introduce bias.

### Summary Measures and synthesis of results

The statistical analyses were performed separately at two different thresholds: initial and more advanced caries lesions. For the more advanced caries lesions threshold, only lesions reaching dentine (when lesion depth was assessed) or cavitated lesions were considered in the studies that the reference standard was performed by direct visual inspection. On the other hand, for the initial caries lesions threshold, we considered all lesions, independent of the lesion depth or of the dental surface integrity (cavitated or not).

The majority of analysis were performed separately considering the different methods, types of teeth and examined dental surfaces. The analyses included:

1. Qualitative description of included studies.
2. “Paired Forest Plot” to report the results of sensitivity and specificity of individual studies for each method combined with the type of tooth and its respective surface (RevMan Version 5.1) [17], [18].
3. Statistical pooling of sensitivity, specificity, diagnostic odds ratio (DOR), positive (PLR) and negative likelihood ratios (NLR), calculated using DerSimonian Laird method (random effects meta-analysis model) using Meta-Disc 1.4 analysis software (Madrid, Spain). Additionally, we summarised these numbers in summary receiver operating characteristic curves (sROC). Graphics were created using the MetaDisc 1.4 Software.
4. Evaluation of individual quality of studies through QUADAS checklist (RevMan Version 5.1).
5. Search for the presence of publication bias through funnel plots, based on the DOR of each study and their respective confidence intervals (CI) (Comprehensive Meta-Analysis Software, Statistical Solutions, Boston, USA).
6. Search for the presence of heterogeneity (inconsistency – *I^2^*) based on DORs of included studies (MetaDisc 1.4 Software).
7. Explore possible explanations for heterogeneity through meta-regressions (MetaDisc 1.4 Software). Meta-regression was performed to compare the effect of methodological differences related to the categories: primary or permanent teeth; clinical or laboratory studies with specimens frozen or not; and type of reference standard methods used (histological, operative intervention or others – visual, tooth separation, radiographic etc.). The statistical significance was set at p<0.05.

We also performed sensitivity analysis with the exclusion of each study sequentially. This analysis was performed to determine the robustness of the results.

## Results

### Study Selection

**Figure 2:**
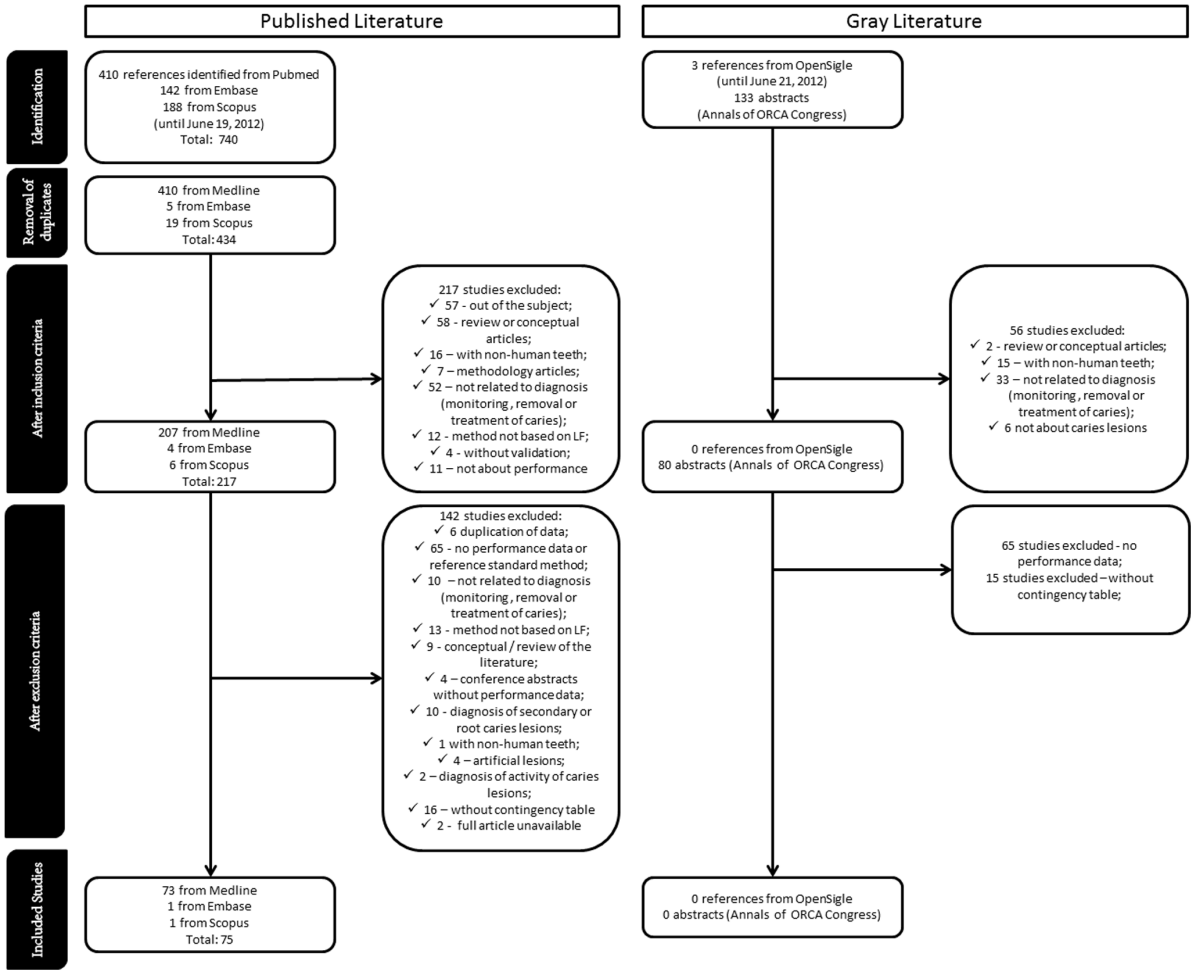
Flow diagram for selection of studies.

Study selection flow is shown in Figure 2. Medline (PubMed), Embase and Scopus searches yielded 740 studies (Figure 2). Using Medline as reference, 306 articles were excluded due to duplication. Thus, the three databases identified 434 unique studies. On the basis of title and abstract, we excluded a further 217 articles. One hundred and forty two articles were excluded after reading full text, due to reasons detailed in Figure 2. This left 75 studies for evaluation. The search of OpenSIGLE and abstracts from Annals of the ORCA Congress yielded 136 investigations (Figure 2), but none were included due to lack of full data about accuracy.

### Study Characteristics

Publication year ranged from 1999 to 2012. The vast majority of studies were conducted in the laboratory using the occlusal surfaces of permanent teeth with a histological reference standard. Most studies were performed using the LF method (DIAGNOdent), followed by studies using LFpen. A summary containing characteristics of each included study is provided in the online supplementary material (Table S1).

### Risk of bias within studies

**Figure 3:**
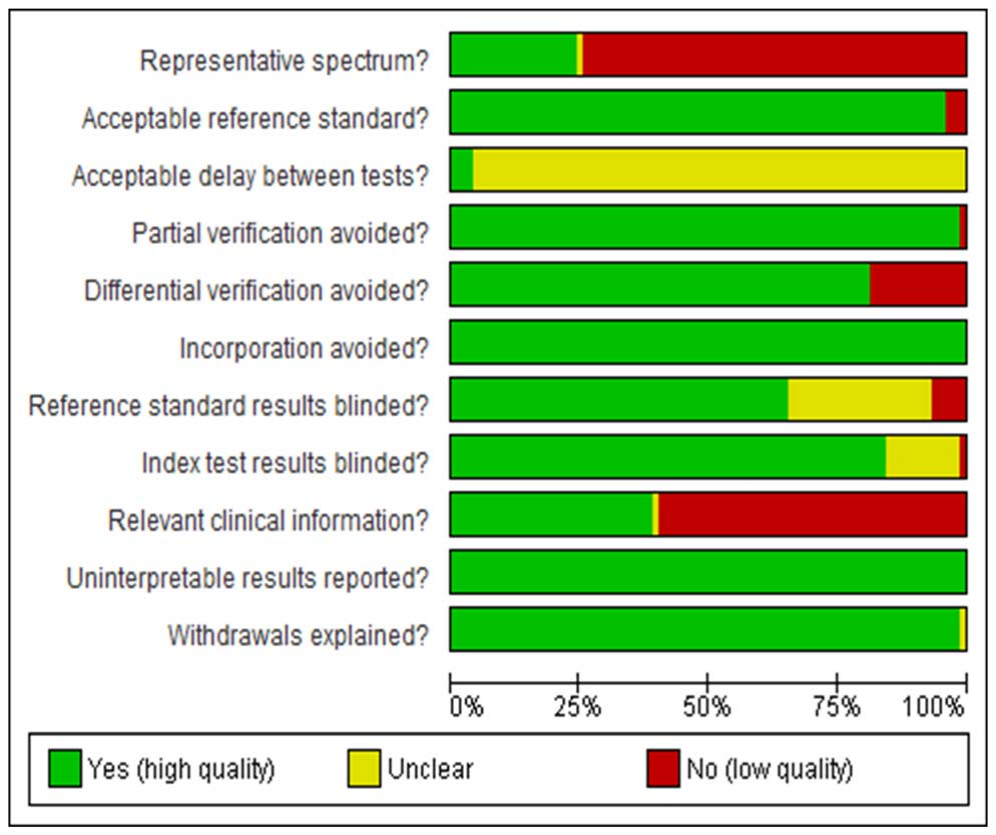
QUADAS graphic. Analysis of study quality considering the Quality assessment of studies of diagnostic performance included in systematic reviews (QUADAS) checklist.

The overview of the QUADAS checklist for all studies demonstrated some differences in terms of study quality. The analysis showed that almost 75% of the studies lacked a representative spectrum of lesion severity. Practically 100% did not specify the time between test and reference standard and nearly 50% did not report relevant clinical information. The great majority of studies used an acceptable reference standard, avoided partial verification and incorporation bias, reported uninterpretable, intermediate or indeterminate results and explained withdraws (Figure 3). Usually, the authors do not mention uninterpretable, intermediate or indeterminate results, and these results are commonly removed from the analysis. However, it is important that these are reported so that the impact of these results on test performance can be determined.

### Results of individual studies

**Figure 4:**
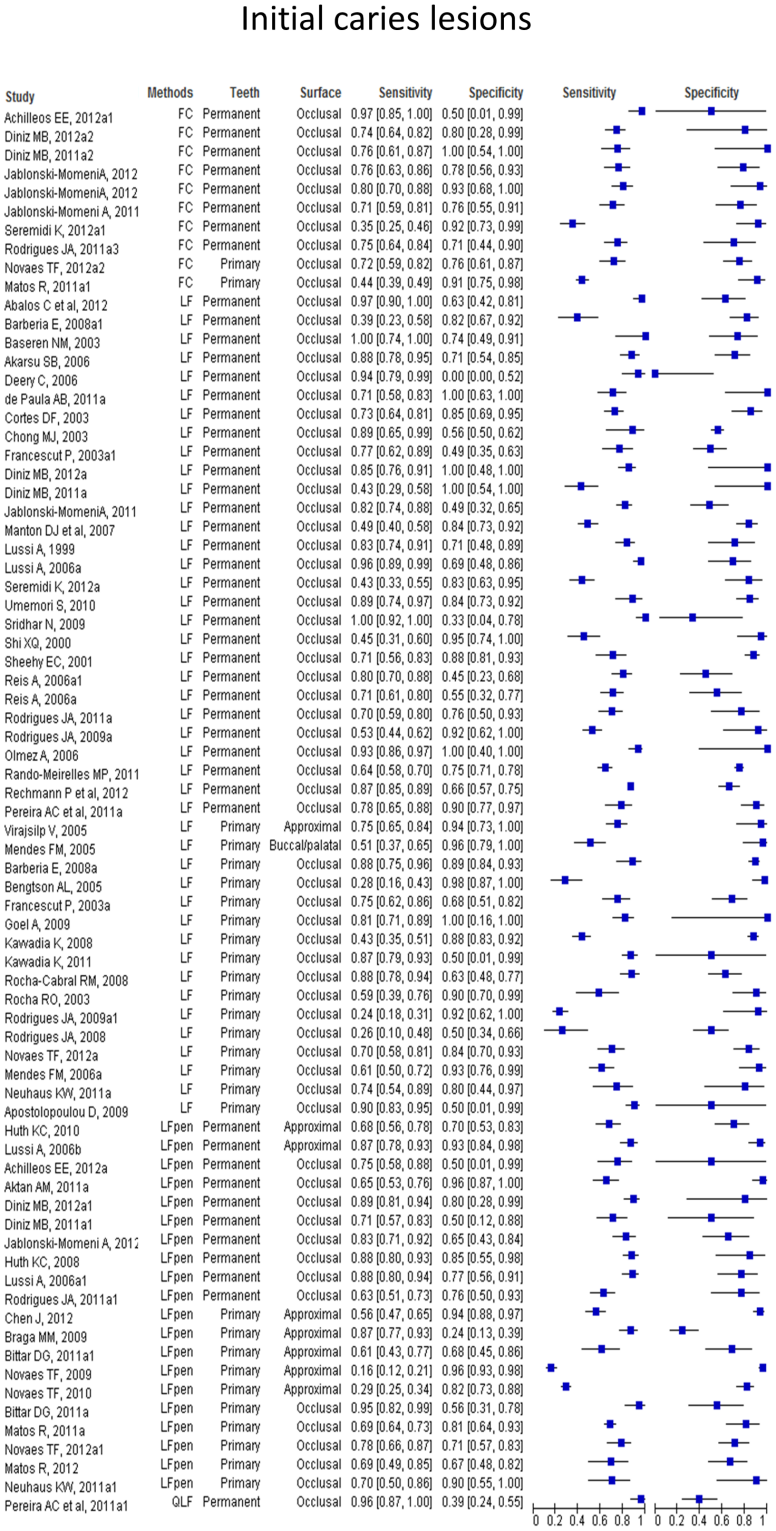
Paired forest plot of the results at initial caries lesions threshold.

**Figure 5:**
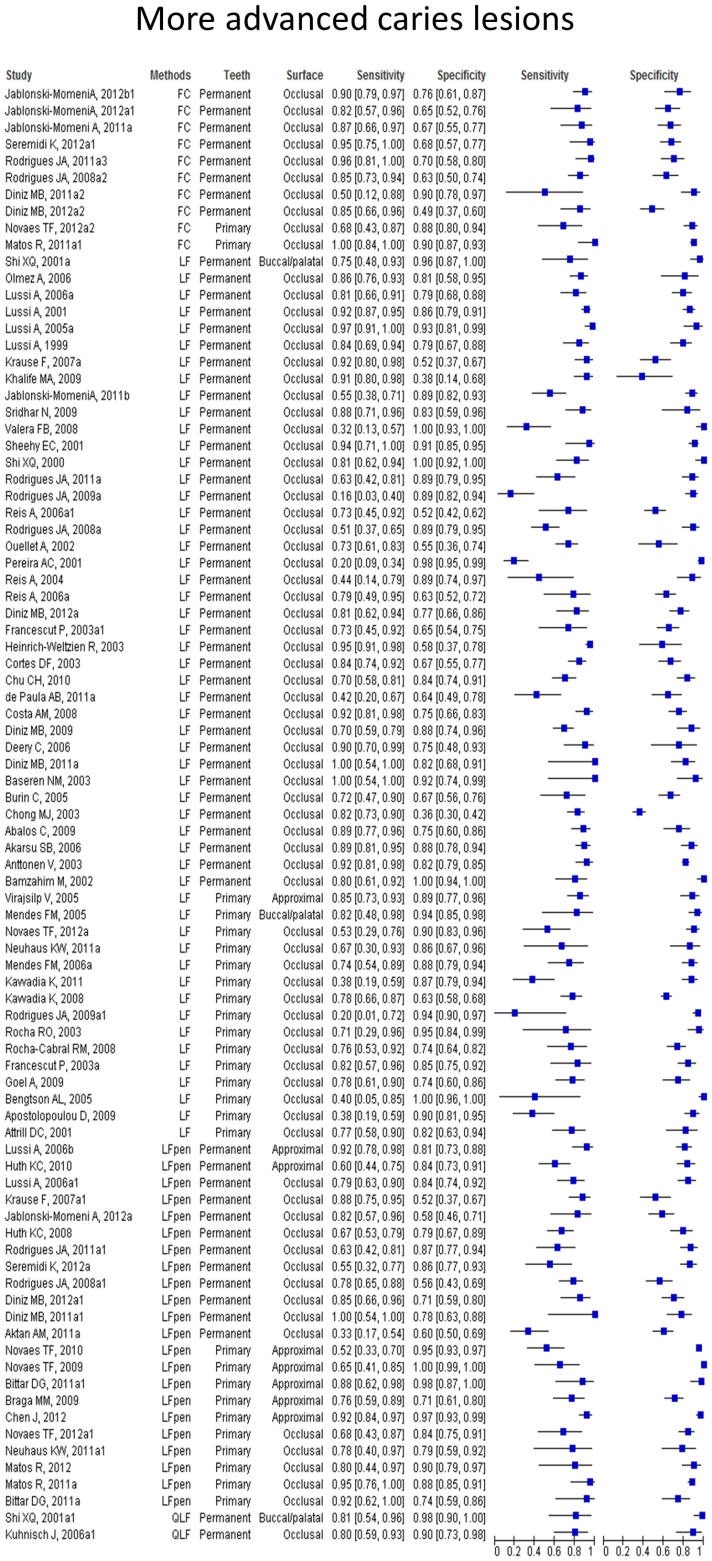
Paired forest plot of the results at more advanced caries lesions threshold.

Paired forest plots show the sensitivities and specificities of each study with their 95% confidence intervals depicted as horizontal lines, grouped by caries detection method, permanent or primary tooth and dental surface tested. We observed a wide range of results across the studies with a tendency to higher sensitivity and specificity values when the methods were used to detect more advanced caries lesions. The paired forest plots of the values of performance at initial caries lesions threshold (Figure 4) and at more advanced caries lesions threshold (Figure 5) are provided.

### Synthesis of results

Pooled sensitivity, specificity, DOR, PLR, NLR, I^2^ and sROC were calculated separately for the method used, type of tooth and dental surface. Within these groups, the area under curves (AUC) of summary ROC analysis provided more adequate description of the study results.

An overall analysis showed that the fluorescence-based methods had similar accuracy for all types of teeth, setting and tooth surfaces. A trend towards better accuracy could be observed at the more advanced caries threshold. A tendency towards higher pooled specificity than the pooled sensitivity could be observed, except for the more advanced lesions threshold on the occlusal surfaces of permanent teeth that showed similar values of sensitivity and specificity.

**Figure 6:**
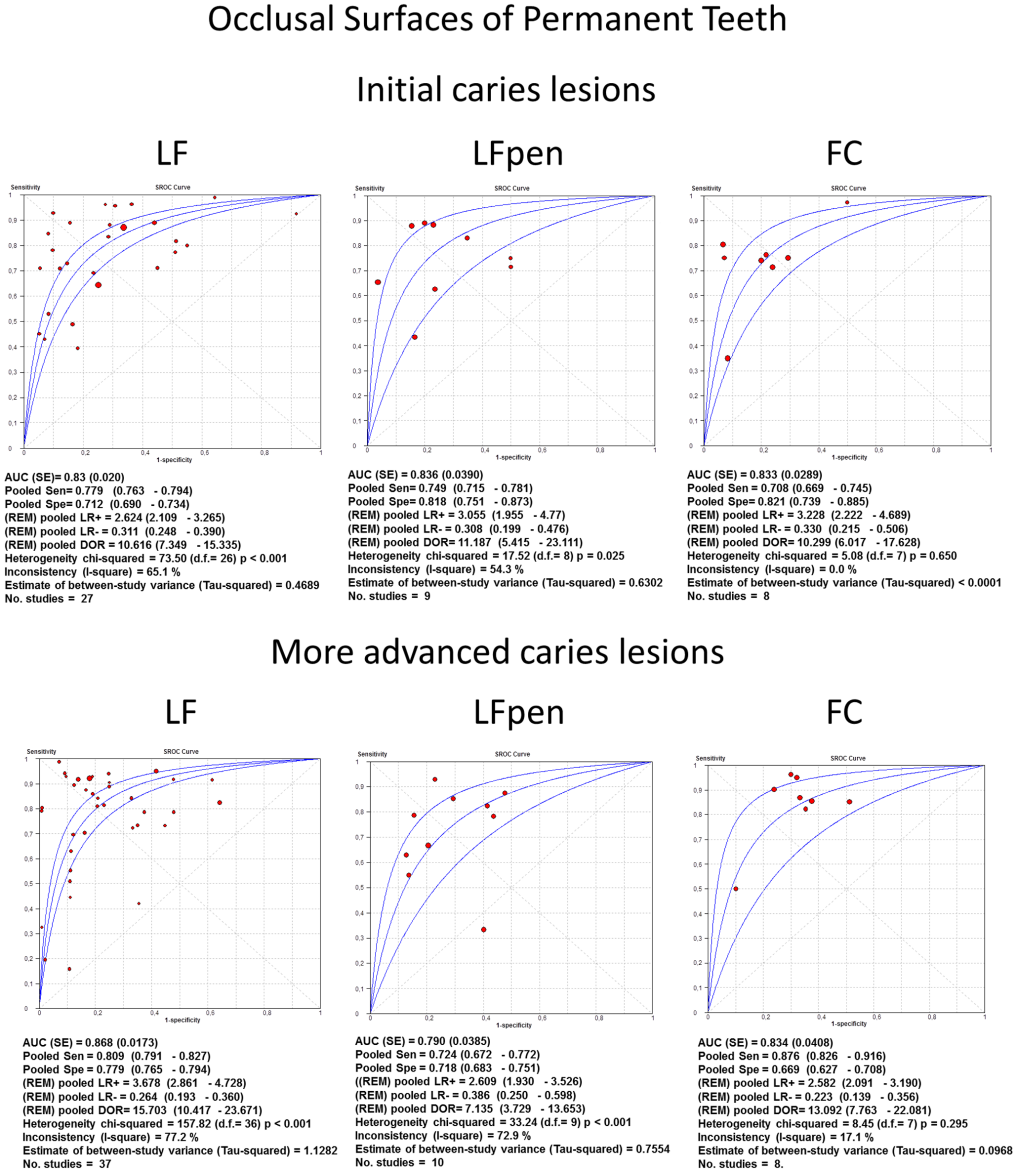
sROC curves of accuracy performed on occlusal surfaces of permanent teeth. Summary Receiver-operating characteristics (sROC) curves and synthesis of the results obtained with studies of accuracy performed on occlusal surfaces of permanent teeth. LF = Laser fluorescence method; LFpen = pen-type LF; FC = Fluorescence camera.

**Figure 7:**
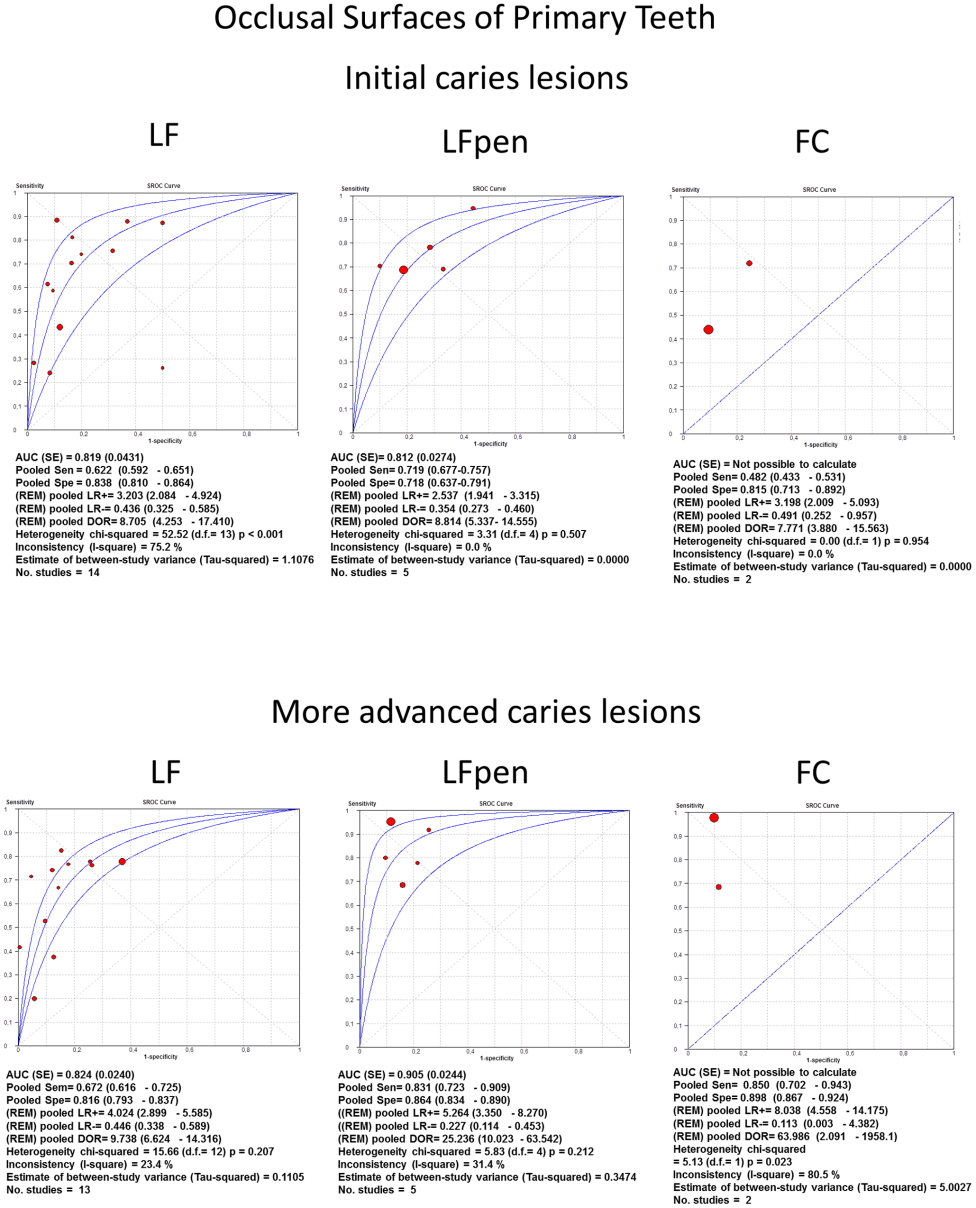
sROC curves of accuracy performed on occlusal surfaces of primary teeth. Summary Receiver-operating characteristics (sROC) curves and synthesis of the results obtained with studies of accuracy performed on occlusal surfaces of primary teeth. LF = Laser fluorescence method; LFpen = pen-type LF; FC = Fluorescence camera.

With regard to the occlusal surfaces of permanent teeth (Figure 6) at initial lesions threshold, the values of pooled sensitivity, specificity, DOR, PLR, NLR, AUC of sROC were pretty similar amongst the three methods (LF, LFpen and FC), while at the more advanced lesions threshold, pooled DOR for LF and FC methods were higher than for LFpen. Considering the occlusal surfaces of primary teeth (Figure 7), the values of pooled sensitivity, specificity, DOR, PLR, NLR, AUC of sROC were again similar among the three methods (LF, LFpen and FC) in detecting initial caries lesions. At more advanced lesions threshold, pooled DOR for LF were the lowest value, LFpen showed an intermediate result and FC method presented the highest value.

**Figure 8:**
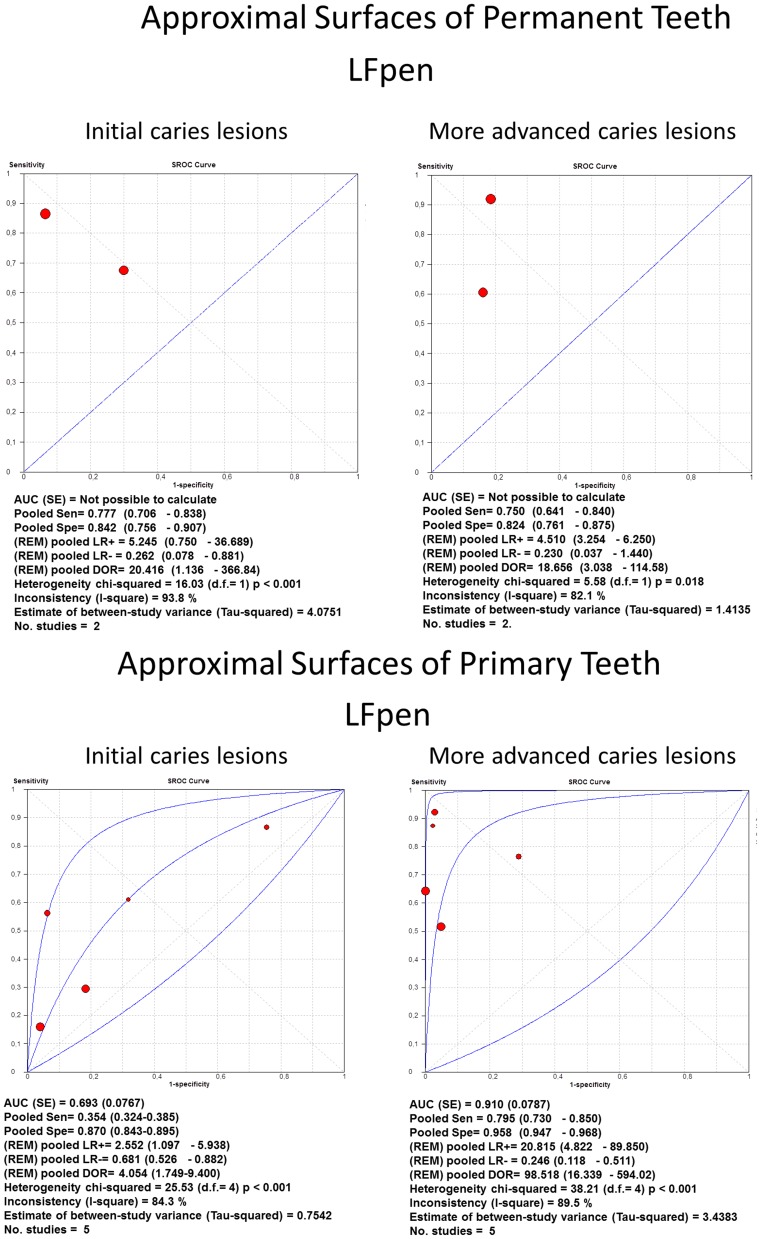
sROC curves of accuracy performed on approximal surfaces. Summary Receiver-operating characteristics (sROC) curves and synthesis of the results obtained with studies about accuracy performed on approximal surfaces. LFpen = pen-type Laser fluorescence method.

For approximal surfaces of both permanent and primary teeth (Figure 8), only LFpen method had sufficient studies to permit a meta-analysis. For permanent teeth, the LFpen showed similar values at both thresholds, whilst for primary teeth, the same method presented higher pooled DOR in detecting more advanced caries lesions.

Only three articles using QLF were included; because of this, we could not perform any meta-analysis. All studies were carried out on permanent teeth. At the non-cavitated lesions threshold, only one study evaluated the accuracy of the method on occlusal surfaces [19]. This reported high sensitivity values at the expense of specificity. Two articles reported the performance at the more advanced lesions threshold. One was conducted on smooth surfaces [20] and the other on occlusal surfaces [21]. They reported high values of both specificity and sensitivity.

Likewise, only two included studies assessed the accuracy of the methods on smooth surfaces. They both used LF device, one on permanent [20] and the other on primary teeth [22]. Furthermore, one of them also evaluated the performance of the QLF on permanent teeth [20]. On both non-cavitated lesions and more advanced lesions thresholds, these reported values of sensitivity lower than those of specificity.

The test chosen for estimating heterogeneity among studies was *I* ^2^. Overall, the studies presented heterogeneity varying from moderate to high. Regarding occlusal surfaces of permanent teeth, the values of *I^2^* were pretty similar between LF and LFpen with moderate heterogeneity at initial caries threshold (65% and 54% respectively), and moderate to high at more advanced lesions threshold (77% and 73% respectively). The FC method presented very low inconsistency at both initial (0%) and more advanced (17%) lesions thresholds. With regard to the occlusal surfaces of primary teeth, *I^2^* values of LFpen and FC methods in detecting initial caries lesions were 0% while LF presented high inconsistency (75%). At more advanced lesions threshold, LF and LFpen showed low to moderate heterogeneity (23% and 31%, respectively) and FC method presented higher heterogeneity (80%). Regarding approximal surfaces of permanent and primary teeth, LFpen method showed high inconsistency in both initial (93% and 84%, respectively) and more advanced (84% and 89%) lesions thresholds.

Heterogeneity analyses were not possible for other situations due to lack of sufficient studies.

### Evidence of publication bias among the studies

**Figure 9:**
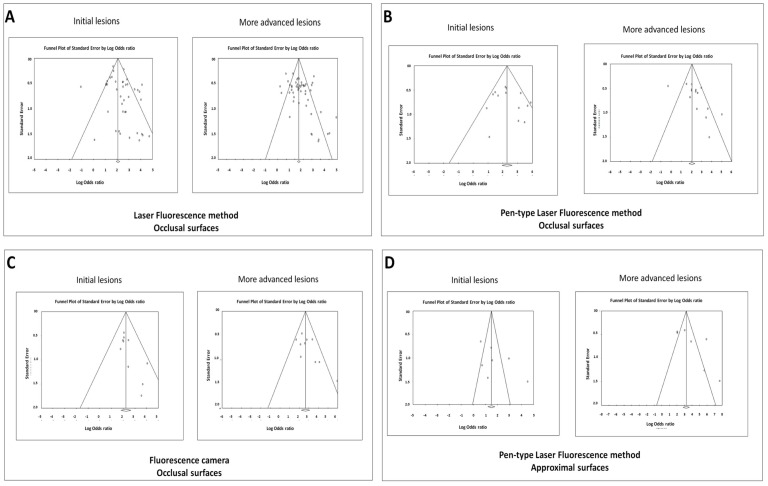
Funnel plots to evaluate evidences of publication bias. Funnel plots plotted to evaluate possibility of publication bias of studies using laser fluorescence method (A), pen-type laser fluorescence (LFpen) method (B) and Fluorescence camera (C) in detecting occlusal caries lesions and LFpen for approximal caries lesions (D).

Funnel plots were performed for each of the methods and tooth surfaces at each lesion severity threshold (Figure 9A to D). We observed evidence of possible publication bias considering the following conditions: LF on occlusal surfaces at both thresholds (Figure 9A); LFpen used on occlusal surfaces only at more advanced caries lesions threshold (Figure 9B); and FC only at initial lesions threshold (Figure 9C). We also observed evidence of publication bias with the LFpen used on approximal surfaces at more advanced caries lesions threshold (Figure 9D).

### Additional analysis

In the sensitivity analysis, we did not observe any statistically significant difference with the exclusion of any study.

Meta-regression analyses were performed to compare the effect of methodological differences related to the different situations: primary vs. permanent teeth; clinical or laboratory setting with specimens frozen or not; and type of reference standard method used (histological, operative intervention or other reference standard methods). Only LF method used on occlusal surfaces of permanent teeth at dentin threshold demonstrated a statistically significant difference comparing in vivo studies and in vitro studies in which the specimens were not frozen (Table 1).

**Table 1:** Meta-regression analysis to compare the effect of differences related to the setting of the studies performed to detect more advanced caries lesions on occlusal surfaces of permanent teeth.

	Laser fluorescence (LF)		Pen-type LF		Fluorescence camera	
	RDOR (95% CI)	p	RDOR (95% CI)	p	RDOR (95% CI)	p
**Clinical**	1.00		1.00		1.00	
**Laboratory**	0.28 (0.12 to 0.66)	0.004	0.35 (0.04 to 2.99)	0.276	3.83 (0.53 to 27.73)	0.132
**Laboratory using frozen teeth**	0.45 (0.12 to 1.67)	0.223	1.27 (0.18 to 8.79)	0.768	2.96 (0.36 to 24.45)	0.227

RDOR = Relative Diagnostic Odds ratio; 95% CI = 95% Confidence interval.

Regarding the type of reference standard, LF method used on occlusal surfaces of permanent teeth at initial caries lesions threshold demonstrated a statistically better performance when other reference standard methods were used compared to the histological examination (Table 2). Studies with the LF method used on occlusal surfaces of permanent teeth at dentin threshold that used operative intervention as reference standard method demonstrated a statistically better performance than studies using histological examination (Table 3). Other meta-regression analyses did not present statistically significant differences and the data were not presented.

**Table 2:** Meta-regression analysis to compare the effect of differences related to reference standard of the studies performed to detect initial caries lesions on occlusal surfaces of permanent teeth.

	Laser fluorescence (LF)		Pen-type LF	
	RDOR (95% CI)	p	RDOR (95% CI)	p
**Histological**	1.00		1.00	
**Operative intervention**	0.91 (0.37 to 2.22)	0.824		
**Others**	4.13 (1.24 to 13.76)	0.023	4.41 (0.19 to 103.92)	0.294

RDOR = Relative Diagnostic Odds ratio; 95% CI = 95% Confidence interval.

**Table 3:** Meta-regression analysis to compare the effect of differences related to reference standard of the studies performed to detect more advanced caries lesions on occlusal surfaces of permanent teeth.

	Laser fluorescence (LF)		Pen-type LF	
	RDOR (95% CI)	p	RDOR (95% CI)	p
**Histological**	1.00		1.00	
**Operative intervention**	3.01 (1.26 to 7.20)	0.015	0.96 (0.12 to 7.77)	0.961
**Others**	0.62 (0.15 to 2.46)	0.483		

RDOR = Relative Diagnostic Odds ratio; 95% CI = 95% Confidence interval.

## Discussion

Systematic reviews are useful methods to present the best existing evidence about a specific question. Clinicians and health care professionals should be aware of the best evidence available to support their clinical practice. Concerning advanced adjunct methods employed to detect dental decay based on fluorescence, a previous systematic review was performed in 2004, but this was limited to the LF method only [23]. Another more recent systematic review has been published, but the authors did not perform a meta-analysis [24]. Our study is the first systematic review of diagnostic methods of caries lesions that has performed a series of meta-analyses and meta-regressions. Thus, we have evaluated empirically the key aspects of different fluorescence based methods used to detect caries lesions, such as the accuracy of these methods, the heterogeneity among the studies, the evidence of publication bias, and if differences in the methodology could interfere with the results of the meta-analysis. Our review is intended to add important information for clinicians to use in order to enable them to make a decision as to the actual usefulness of the fluorescence-based methods.

The review search was limited to four laser fluorescence methods which were those that were reported most in the literature: LF, LFpen, FC and QLF. We observed that all devices showed similar results about accuracy. These results were observed independent of the tooth type or dental surfaces examined.

The findings with regard to the similar accuracy of the devices is to be expected because although of different designs and working with different light sources and wavelengths, these methods are based on the fact that carious tissue fluoresces differently to sound surfaces when excited by light at a certain wavelength range. The only significant difference is that QLF predominantly measures the loss of intrinsic fluorescence of the dental enamel caused by demineralization and the other methods (LF, LFpen and FC) are based on the alterations (increase) in fluoresce of carious tissues due to the presence of bacterial metabolites [8], [25], [26].

Some significant differences were observed. For example, studies have suggested that the results obtained with the original LF cannot necessarily be extrapolated to those obtained with the new LFpen or with the FC [26], [27]. This assertion is because the LFpen device tends to give higher readings than the LF; hence different cut-off points should be considered for the different devices. In our study, we found similar performance among the methods probably because the meta-analysis tends to adjust for these differences in the cut-off points.

The most commonly used indicators of diagnostic performance have been sensitivity and specificity. We could see a trend of pooled specificity being greater than the pooled sensitivity, except for the dentine threshold on the occlusal surfaces of permanent teeth. This is important as most new lesions in young patients occur on the occlusal surface and the dentine threshold may be used by some to base operative intervention on. Having a lower specificity on this surface at this threshold could lead to overprescription and unnecessary treatment. This tendency of higher specificity and lower sensitivity at the initial threshold was also observed in a previous systematic review considering only the LF [23]; however, our results on primary teeth showed a different pattern of results of this previous review. Specificity values were higher than the sensitivities at both thresholds. Nevertheless, when the results of different studies are pooled, the threshold effect usually occurs, as both sensitivity and specificity parameters are not independent [28].

Thus, the best indicator of accuracy is the DOR, which is a parameter that combines diagnostic values of accuracy in a single value. DOR does not suffer influence of the threshold effect among the studies. Considering this parameter, a trend of better performance at the more advanced caries lesions threshold could be observed. This pattern has been observed in several individual studies using fluorescence-based methods in detecting occlusal [10], [29]–[32] and approximal [9], [33]–[35] caries lesions.

Regarding heterogeneity, *I* ^2^ describes the percentage of total variation across the studies which is due to heterogeneity rather than chance. A value of 0% indicates no observed heterogeneity and larger values show increasing heterogeneity. It is not always appropriate categorizing *I^2^*, but it is possible to assign ranges of values adjectives such as low, moderate and high values of *I^2^* to 25%, 50% and 75% respectively [36]. In the present study, we observed inconsistency ranging from moderate to high in the analyses; however, as systematic reviews bring together studies that are different in several aspects, heterogeneity is expected. Research about the inconsistency of the studies involves more than just quantifying it, but to identify differences in clinical and methodological aspects [36]. There are different approaches suggested to deal with the sources of heterogeneity described in the literature [37]: (1) Ignore the heterogeneity using fixed effect models; (2) Consider the heterogeneity using random models; and (3) Explore the heterogeneity through subgroup analysis or meta-regression.

Concerning the meta-regressions performed in our study, we compared the effect of methodological differences related to the important aspects of the studies: studies using primary or permanent teeth; clinical or laboratory setting; and differences related to the reference standard method used. Considering the setting, we also divided the laboratory investigations into studies which used frozen specimens or not. Previous research has demonstrated that the best method to store texted teeth in LF studies is to freeze them at −20°C [38]. We found that only LF method used on occlusal surfaces of permanent teeth with the more advanced caries lesions threshold demonstrated a statistically significant difference between the clinical setting and in vitro studies whether the specimen was frozen or not [38]. Surprisingly, with regard to the type of teeth, differences in the accuracy between primary and permanent teeth were not observed, although important anatomical and compositional differences exist between them [39], [40].

Regarding the reference standard methods, at initial caries lesions threshold, we observed a better performance in studies using other reference standards when compared to histological examination. Probably, this finding was because other reference standard methods usually incorporate visual inspection to detect initial lesions. Other finding of our study was that studies using LF at more advanced caries lesions threshold with operative intervention as reference standard presented better performance than those with histological validation. This difference could be explained by the existence of differentiated or partial verification of the sample [41]. In this case, a differential verification could cause a given quantity of lesions is assumed to be sound by visual inspection and is not evaluated by operative intervention. Thus, there would be an overestimation of the test accuracy.

Another way to evaluate possible sources of heterogeneity is through quality analysis. The QUADAS checklist showed that almost 75% of the included studies lacked a representative spectrum of lesion severity, mainly because the vast majority of articles were performed under laboratory conditions. Further, some clinical studies did not have a representative spectrum because they chose specific teeth (third molars or periodontally compromised teeth). For the same reasons, over 50% of the studies did not give relevant clinical information. When the spectrum of lesions or other type of biases are present, a significant overestimation in the accuracy is expected [41]. Therefore, the authors should design research to avoid these possible biases, mainly spectrum bias.

The publication bias has been defined as the tendency on the part of investigators to submit, and or the reviewers and editors to accept, manuscripts based on the direction or strength of the study findings [28]. There is a tendency to publish the strongest and most positive studies, with negative experiments with small sample size having less chance to be published [42]. Most of the funnel plots obtained in our study indicated evidence of publication bias for different reviewed methods and study conditions.

Although some studies have shown that the exclusion of articles published in other languages does not seem to bias systematic reviews [43], [44], we included non-english manuscripts in our review. Six articles were fully analyzed; however, they failed in reporting some important data and were not included in the meta-analysis. Regarding the databases searched, it is known that a survey based on searches carried out only in the MEDLINE database is not considered appropriate for systematic reviews and may lead to the occurrence of bias due to missing studies [45]–[47]. Thus, we attempted to minimize this limitation by searching for articles in other sources, including gray literature. Unfortunately, this search provided no additional studies in our review, since abstracts lacked the data needed to build the 2×2 tables required for calculation of the necessary statistical parameters. This problem can be solved if abstracts of future primary research include a contingency table or the sample size and caries prevalence of their sample.

We observed in our systematic review that the fluorescence-based methods presented similar results concerning the accuracy, heterogeneity, quality of the studies and publication bias. However, despite the similarity among these advanced methods, the authors should take into account the accuracy of additional methods compared with that of visual inspection. The pooled sensitivities in detecting more advanced caries lesions obtained with the different fluorescence-based methods tended to be higher than those obtained with visual inspection in clinical studies of occlusal surfaces [6]. On the other hand, the pooled specificities were likely to be lower than those obtained with clinical examination [31], [48], [49]. This pattern was more evident on approximal surface studies [33], [35]. Considering the overall accuracy, however, no evident differences can be observed. Therefore, the actual improvement of the accuracy using the adjunct methods in the caries detection strategy is unclear. In fact, two recent clinical studies about caries detection strategies have contested the benefits of the adjunct methods compared to the visual inspection performed alone [50], [51]. A systematic review with meta-analysis about visual inspection for detection of caries lesions should therefore be performed to evaluate the overall accuracy of the method and to permit comparisons to be made with other adjunct caries detection methods.

In conclusion, despite the heterogeneity of the studies and evidence of publication bias, all the fluorescence-based methods showed similar accuracy in detecting occlusal and approximal caries lesions, on both primary and permanent teeth. The performance tended to be better in detecting more advanced caries lesions. The majority of studies included in this review were performed under laboratory conditions or with an inappropriate spectrum of patients/lesions which limits the extrapolation of the actual usefulness of these methods to the clinical situation.

## Supporting Information

Table S1 **Summary of characteristics of included studies.**
(DOCX)

Table S2 **PRISMA (Preferred Reporting Items for Systematic Reviews and Meta-Analyses) check list.**
(DOCX)

## References

[1] Bonecker M, Ardenghi TM, Oliveira LB, Sheiham A, Marcenes W (2010) Trends in dental caries in 1- to 4-year-old children in a Brazilian city between 1997 and 2008. Int J Paediatr Dent 20: 125–131. 20384827 10.1111/j.1365-263X.2009.01030.x

[2] Ekstrand KR (2004) Improving clinical visual detection–potential for caries clinical trials. J Dent Res 83 Spec No C: C67–71. 15286125 10.1177/154405910408301s13

[3] Nyvad B (2004) Diagnosis versus detection of caries. Caries Res 38: 192–198. 15153688 10.1159/000077754

[4] Braga MM, Mendes FM, Ekstrand KR (2010) Detection activity assessment and diagnosis of dental caries lesions. Dent Clin North Am 54: 479–493. 20630191 10.1016/j.cden.2010.03.006

[5] Pitts NB, Boyles J, Nugent ZJ, Thomas N, Pine CM (2003) The dental caries experience of 5-year-old children in England and Wales. Surveys co-ordinated by the British Association for the Study of Community Dentistry in 2001/2002. Community Dent Health 20: 45–54. 12688604

[6] Bader JD, Shugars DA, Bonito AJ (2002) A systematic review of the performance of methods for identifying carious lesions. J Public Health Dent 62: 201–213. 12474624 10.1111/j.1752-7325.2002.tb03446.x

[7] Angmar-Mansson B, ten Bosch JJ (2001) Quantitative light-induced fluorescence (QLF): a method for assessment of incipient caries lesions. Dentomaxillofac Radiol 30: 298–307. 11641727 10.1038/sj/dmfr/4600644

[8] Hibst R, Paulus R, Lussi A (2001) Detection of occlusal caries by laser fluorescence: Basic and clinical investigations. Medical Laser Application 16: 205–213.

[9] Lussi A, Hack A, Hug I, Heckenberger H, Megert B, et al (2006) Detection of approximal caries with a new laser fluorescence device. Caries Res 40: 97–103. 16508265 10.1159/000091054

[10] Lussi A, Hellwig E (2006) Performance of a new laser fluorescence device for the detection of occlusal caries in vitro. Journal of Dentistry 34: 467–471. 16431009 10.1016/j.jdent.2005.11.002

[11] Achilleos EE, Rahiotis C, Kakaboura A, Vougiouklakis G (2012) Evaluation of a new fluorescence-based device in the detection of incipient occlusal caries lesions. Lasers Med Sci 10.1007/s10103-012-1111-6 22576667

[12] Moher D, Liberati A, Tetzlaff J, Altman DG (2009) Preferred reporting items for systematic reviews and meta-analyses: the PRISMA statement. PLoS Med 6: e1000097. 19621072 10.1371/journal.pmed.1000097 PMC2707599

[13] Deville WL, Bezemer PD, Bouter LM (2000) Publications on diagnostic test evaluation in family medicine journals: an optimal search strategy. J Clin Epidemiol 53: 65–69. 10693905 10.1016/s0895-4356(99)00144-4

[14] Nelemans PJ, Leiner T, de Vet HC, van Engelshoven JM (2000) Peripheral arterial disease: meta-analysis of the diagnostic performance of MR angiography. Radiology 217: 105–114. 11012430 10.1148/radiology.217.1.r00oc11105

[15] Whiting P, Rutjes AW, Reitsma JB, Bossuyt PM, Kleijnen J (2003) The development of QUADAS: a tool for the quality assessment of studies of diagnostic accuracy included in systematic reviews. BMC Med Res Methodol 3: 25. 14606960 10.1186/1471-2288-3-25 PMC305345

[16] Reitsma J, Rutjes A, Whiting P, Vlassov V, Leeflang M, et al.. ( 2009) Chapter 9: Assessing methodological quality. In: Deeks J, Bossuyt P, Gatsonis C, editors. Cochrane Handbook for Systematic Reviews of Diagnostic Test Accuracy Version 100: The Cochrane Collaboration.

[17] Leeflang MM, Deeks JJ, Gatsonis C, Bossuyt PM (2008) Systematic reviews of diagnostic test accuracy. Ann Intern Med 149: 889–897. 19075208 10.7326/0003-4819-149-12-200812160-00008 PMC2956514

[18] Macaskill P, Gatsonis C, Deeks J, Harbord R, Takwoingi Y (2010) Chapter 10: Analysing and Presenting Results. In: Deeks J, Bossuyt P, Gatsonis C, editors. Cochrane Handbook for Systematic Reviews of Diagnostic Test Accuracy Version 10: The Cochrane Collaboration.

[19] Pereira AC, Eggertsson H, González-Cabezas C, Zero DT, Eckert GJ, et al (2011) Quantitative light-induced fluorescence (QLF) in relation to other technologies and conventional methods for detecting occlusal caries in permanent teeth. Brazilian Journal of Oral Sciences 10: 27–32.

[20] Shi XQ, Tranaeus S, Angmar-Mansson B (2001) Comparison of QLF and DIAGNOdent for quantification of smooth surface caries. Caries Res 35: 21–26. 11125192 10.1159/000047426

[21] Kuhnisch J, Ifland S, Tranaeus S, Angmar-Mansson B, Hickel R, et al (2006) Establishing quantitative light-induced fluorescence cut-offs for the detection of occlusal dentine lesions. Eur J Oral Sci 114: 483–488. 17184229 10.1111/j.1600-0722.2006.00404.x

[22] Mendes FM, Siqueira WL, Mazzitelli JF, Pinheiro SL, Bengtson AL (2005) Performance of DIAGNOdent for detection and quantification of smooth-surface caries in primary teeth. J Dent 33: 79–84. 15652172 10.1016/j.jdent.2004.10.010

[23] Bader JD, Shugars DA (2004) A systematic review of the performance of a laser fluorescence device for detecting caries. J Am Dent Assoc 135: 1413–1426. 15551982 10.14219/jada.archive.2004.0051

[24] Twetman S, Axelsson S, Dahlen G, Espelid I, Mejare I, et al (2012) Adjunct methods for caries detection: A systematic review of literature. Acta Odontol Scand 10.3109/00016357.2012.690448 22630355

[25] Buchalla W (2005) Comparative fluorescence spectroscopy shows differences in noncavitated enamel lesions. Caries Res 39: 150–156. 15741729 10.1159/000083162

[26] De Benedetto MS, Morais CC, Novaes TF, de Almeida Rodrigues J, Braga MM, et al (2011) Comparing the reliability of a new fluorescence camera with conventional laser fluorescence devices in detecting caries lesions in occlusal and smooth surfaces of primary teeth. Lasers Med Sci 26: 157–162. 20157753 10.1007/s10103-010-0757-1

[27] Kuhnisch J, Bucher K, Henschel V, Hickel R (2007) Reproducibility of DIAGNOdent 2095 and DIAGNOdent Pen measurements: results from an in vitro study on occlusal sites. Eur J Oral Sci 115: 206–211. 17587296 10.1111/j.1600-0722.2007.00441.x

[28] Cota GF, de Sousa MR, Demarqui FN, Rabello A (2012) The diagnostic accuracy of serologic and molecular methods for detecting visceral leishmaniasis in HIV infected patients: meta-analysis. PLoS Negl Trop Dis 6: e1665. 22666514 10.1371/journal.pntd.0001665 PMC3362615

[29] Lussi A, Megert B, Longbottom C, Reich E, Francescut P (2001) Clinical performance of a laser fluorescence device for detection of occlusal caries lesions. Eur J Oral Sci 109: 14–19. 11330928 10.1034/j.1600-0722.2001.109001014.x

[30] Francescut P, Lussi A (2003) Correlation between fissure discoloration, Diagnodent measurements, and caries depth: an in vitro study. Pediatr Dent 25: 559–564. 14733470

[31] Matos R, Novaes TF, Braga MM, Siqueira WL, Duarte DA, et al (2011) Clinical performance of two fluorescence-based methods in detecting occlusal caries lesions in primary teeth. Caries Res 45: 294–302. 21625126 10.1159/000328673

[32] Seremidi K, Lagouvardos P, Kavvadia K (2012) Comparative in vitro validation of VistaProof and DIAGNOdent pen for occlusal caries detection in permanent teeth. Oper Dent 37: 234–245. 22166109 10.2341/10-326-L

[33] Novaes TF, Matos R, Braga MM, Imparato JC, Raggio DP, et al (2009) Performance of a pen-type laser fluorescence device and conventional methods in detecting approximal caries lesions in primary teeth–in vivo study. Caries Res 43: 36–42. 19136830 10.1159/000189705

[34] Braga MM, Morais CC, Nakama RC, Leamari VM, Siqueira WL, et al (2009) In vitro performance of methods of approximal caries detection in primary molars. Oral Surg Oral Med Oral Pathol Oral Radiol Endod 108: e35–41. 10.1016/j.tripleo.2009.05.017 19778733

[35] Novaes TF, Matos R, Raggio DP, Imparato JC, Braga MM, et al (2010) Influence of the discomfort reported by children on the performance of approximal caries detection methods. Caries Res 44: 465–471. 20861630 10.1159/000320266

[36] Higgins JP, Thompson SG, Deeks JJ, Altman DG (2003) Measuring inconsistency in meta-analyses. BMJ 327: 557–560. 12958120 10.1136/bmj.327.7414.557 PMC192859

[37] Dinnes J, Deeks J, Kirby J, Roderick P (2005) A methodological review of how heterogeneity has been examined in systematic reviews of diagnostic test accuracy. Health Technol Assess 9: 1–113, iii. 10.3310/hta9120 15774235

[38] Francescut P, Zimmerli B, Lussi A (2006) Influence of different storage methods on laser fluorescence values: a two-year study. Caries Res 40: 181–185. 16707864 10.1159/000092223

[39] Mortimer KV (1970) The relationship of deciduous enamel structure to dental disease. Caries Res 4: 206–223. 5270190 10.1159/000259643

[40] Shellis RP (1984) Relationship between human enamel structure and the formation of caries-like lesions in vitro. Arch Oral Biol 29: 975–981. 6598367 10.1016/0003-9969(84)90144-4

[41] Lijmer JG, Mol BW, Heisterkamp S, Bonsel GJ, Prins MH, et al (1999) Empirical evidence of design-related bias in studies of diagnostic tests. JAMA 282: 1061–1066. 10493205 10.1001/jama.282.11.1061

[42] de Jong MC, Genders TS, van Geuns RJ, Moelker A, Hunink MG (2012) Diagnostic performance of stress myocardial perfusion imaging for coronary artery disease: a systematic review and meta-analysis. Eur Radiol 22: 1881–1895. 22527375 10.1007/s00330-012-2434-1 PMC3411304

[43] Juni P, Holenstein F, Sterne J, Bartlett C, Egger M (2002) Direction and impact of language bias in meta-analyses of controlled trials: empirical study. Int J Epidemiol 31: 115–123. 11914306 10.1093/ije/31.1.115

[44] Moher D, Pham B, Klassen TP, Schulz KF, Berlin JA, et al (2000) What contributions do languages other than English make on the results of meta-analyses? J Clin Epidemiol 53: 964–972. 11004423 10.1016/s0895-4356(00)00188-8

[45] de Vet H, Eisinga A, Riphagen I, Aertgeerts B, Pewsner D (2008) Chapter 7: Searching for Studies. In: Eisinga A, editor. Cochrane Handbook for Systematic Reviews of Diagnostic Test Accuracy Version 04 [updated September 2008]: The Cochrane Collaboration.

[46] Golder S, McIntosh HM, Duffy S, Glanville J (2006) Developing efficient search strategies to identify reports of adverse effects in MEDLINE and EMBASE. Health Info Libr J 23: 3–12. 10.1111/j.1471-1842.2006.00634.x 16466494

[47] Whiting P, Westwood M, Burke M, Sterne J, Glanville J (2008) Systematic reviews of test accuracy should search a range of databases to identify primary studies. J Clin Epidemiol 61: 357–364. 18313560 10.1016/j.jclinepi.2007.05.013

[48] Chen J, Qin M, Ma W, Ge L (2012) A clinical study of a laser fluorescence device for the detection of approximal caries in primary molars. Int J Paediatr Dent 22: 132–138. 21951216 10.1111/j.1365-263X.2011.01180.x

[49] Diniz MB, Boldieri T, Rodrigues JA, Santos-Pinto L, Lussi A, et al (2012) The performance of conventional and fluorescence-based methods for occlusal caries detection: an in vivo study with histologic validation. J Am Dent Assoc 143: 339–350. 22467694 10.14219/jada.archive.2012.0176

[50] Baelum V, Hintze H, Wenzel A, Danielsen B, Nyvad B (2012) Implications of caries diagnostic strategies for clinical management decisions. Community Dent Oral Epidemiol 40: 257–266. 22103270 10.1111/j.1600-0528.2011.00655.x

[51] Mendes FM, Novaes TF, Matos R, Bittar DG, Piovesan C, et al (2012) Radiographic and Laser Fluorescence Methods Have No Benefits for Detecting Caries in Primary Teeth. Caries Res 46: 536–543. 22907166 10.1159/000341189

