# Supplementary material for: Fluorescence-Based Methods for Detecting Caries Lesions: Systematic Review, Meta-Analysis and Sources of Heterogeneity
Source: PLoS One. 2013 Apr 4;8(4):e60421. doi: 10.1371/journal.pone.0060421 (PMC3617206; doi:10.1371/journal.pone.0060421)
Supplement: Table S1 — Summary of characteristics of included studies. (DOCX) [file pone.0060421.s001.docx]

**Supporting Information**

**Table 1S**

| **Study ID** | **Database** | **N** | **D1 Sn** | **D1 Sp** | **D3 Sn** | **D3 Sp** | **method** | **primary/ permanent** | **Tooth surface** | **in vitro/ in vivo** | **Reference standard** | **cut-off value** |
| --- | --- | --- | --- | --- | --- | --- | --- | --- | --- | --- | --- | --- |
| Abalos et al. 2012 [[1](#_ENREF_1)] | Medline | 96 | 0.97 | 0.63 |  |  | LF | permanent | occlusal | vivo | operative intervention | Enamel (>15) |
| Achilleos et al. 2012a  [[2](#_ENREF_2)] | Medline | 38 | 0.75 | 0.5 |  |  | LFpen | permanent | occlusal | vitro without frozen | histological | Sound (0–13); Enamel (14–29); Dentinal (>30) |
| Achilleos et al. 2012a1  [[2](#_ENREF_2)] | Medline | 38 | 0.97 | 0.5 |  |  | FC | permanent | occlusal | vitro without frozen | histological | Sound (0- 0.9); Enamel (1.0–2.0); Dentinal (2.0–5.0) |
| Chen et al. 2012  [[3](#_ENREF_3)] | Medline | 256 | 0.56 | 0.94 | 0.92 | 0.97 | LFpen | primary | approximal | vivo | operative intervention | Sound (≤ 7); Enamel (8-16); Dentinal (≥ 17) |
| Diniz et al. 2012a  [[4](#_ENREF_4)] | Medline | 105 | 0.85 | 1 | 0.81 | 0.77 | LF | permanent | occlusal | vivo | histological | Sound (0-4); Enamel (5-27); Dentinal (28-99) |
| Diniz et al. 2012a1  [[4](#_ENREF_4)] | Medline | 105 | 0.89 | 0.8 | 0.85 | 0.71 | LFpen | permanent | occlusal | vivo | histological | Sound (0-4); Enamel (5-32); Dentinal (33-99) |
| Diniz et al. 2012a2  [[4](#_ENREF_4)] | Medline | 105 | 0.74 | 0.8 | 0.85 | 0.49 | FC | permanent | occlusal | vivo | histological | Sound (0.0-1.2), Enamel (1.3), Dentinal (1.4-5.0) |
| Jablonski-Momeni et al. 2012a  [[5](#_ENREF_5)] | Medline | 82 | 0.83 | 0.65 | 0.82 | 0.59 | LFpen | permanent | occlusal | vitro without frozen | histological | Sound (≤ 5); Enamel (6-13); Dentinal (≥ 17) |
| Jablonski-Momeni et al. 2012a1 [[5](#_ENREF_5)] | Medline | 82 | 0.76 | 0.78 | 0.82 | 0.65 | FC | permanent | occlusal | vitro without frozen | histological | Enamel (1.2/1.3); Dentinal (1.3/1.4) |
| Jablonski-Momeni et al. 2012b1  [[6](#_ENREF_6)] | Medline | 97 | 0.81 | 0.93 | 0.90 | 0.77 | FC | permanent | occlusal | vitro without frozen | histological | Sound (0.0–0.90); Enamel (>0.9–1.50); Dentinal (>2.0–2.50) |
| Matos et al. 2012 [[7](#_ENREF_7)] | Medline | 62 | 0.69 | 0.67 | 0.78 | 0.90 | LFpen | primary | occlusal | vitro frozen | histological | Sound (≤ 8); Enamel (≥ 9); Dentinal (≥ 31) |
| Novaes et al. 2012a  [[8](#_ENREF_8)] | Medline | 113 | 0.703 | 0.837 | 0.526 | 0.904 | LF | primary | occlusal | vitro frozen | histological | Sound (≤ 8); Enamel (> 8); Dentinal (≥ 24) |
| Novaes et al. 2012a1  [[8](#_ENREF_8)] | Medline | 113 | 0.781 | 0.714 | 0.684 | 0.840 | LFpen | primary | occlusal | vitro frozen | histological | Sound (≤ 9); Enamel (> 9); Dentinal (≥ 31) |
| Novaes et al. 2012a2  [[8](#_ENREF_8)] | Medline | 113 | 0.719 | 0.755 | 0.684 | 0.883 | FC | primary | occlusal | vitro frozen | histological | Sound (0–1.1); Enamel (> 1.1) |
| Rechmann et al. 2012  [[9](#_ENREF_9)] | Medline | 1034 | 0.87 | 0.66 |  |  | LF | permanent | occlusal | vivo | visual | Enamel (>15) |
| Seremidi et al. 2012a  [[10](#_ENREF_10)] | Medline | 107 | 0.432 | 0.814 | 0.550 | 0.862 | LFpen | permanent | occlusal | vitro without frozen | histological | Sound (≤ 8); Enamel (9-24); Dentinal (≥ 44) |
| Seremidi et al. 2012a1  [[10](#_ENREF_10)] | Medline | 107 | 0.351 | 0.929 | 0.950 | 0.678 | FC | permanent | occlusal | vitro without frozen | histological | Enamel (1.30); Dentinal (1.59) |
| Aktan et al. 2011a  [[11](#_ENREF_11)] | Medline | 129 | 0.65 | 0.97 | 0.33 | 0.60 | LFpen | permanent | occlusal | vitro without frozen | histological | Sound (0–13); Enamel (14–20); Dentinal (21–99) |
| Bittar et al. 2011a  [[12](#_ENREF_12)] | Medline | 55 | 0.946 | 0.556 | 0.917 | 0.744 | LFpen | primary | occlusal | vitro frozen | histological | Sound (<9); Enamel (>9); Dentinal (>31) |
| Bittar et al. 2011a1 [[12](#_ENREF_12)] | Medline | 58 | 0.611 | 0.682 | 0.846 | 0.978 | LFpen | primary | approximal | vitro frozen | histological | Sound (<8); Enamel (>8); Dentinal (>30) |
| de Paula et al. 2011a  [[13](#_ENREF_13)] | Medline | 64 | 0.72 | 1.00 | 0.42 | 0.65 | LF | permanent | occlusal | vitro without frozen | histological | Sound (0–10); Enamel (11–20); Dentinal (21–99) |
| Diniz et al. 2011a  [[14](#_ENREF_14)] | Medline | 55 | 0.42 | 0.92 | 1.0 | 0.81 | LF | permanent | occlusal | vitro frozen | histological | Sound (0–15); Enamel (16–25); Dentinal (≥25) |
| Diniz et al. 2011a1  [[14](#_ENREF_14)] | Medline | 55 | 0.71 | 0.58 | 0.92 | 0.77 | LFpen | permanent | occlusal | vitro frozen | histological | Sound (0–10), Enamel (11–34),Dentinal (≥34) |
| Diniz et al. 2011a2  [[14](#_ENREF_14)] | Medline | 55 | 0.76 | 1.00 | 0.58 | 0.89 | FC | permanent | occlusal | vitro frozen | histological | Sound (0–1.1), Enamel (1.2–1.7), Dentinal (≥1.7) |
| Jablonski-Momeni et al. 2011a  [[15](#_ENREF_15)] | Medline | 98 | 0.71 | 0.76 | 0.87 | 0.67 | FC | permanent | occlusal | vitro without frozen | histological | Sound (<1.2); Enamel (1.2 / 1.3); Dentinal (1.3 / 1.4) |
| Jablonski-Momeni et al. 2011b  [[16](#_ENREF_16)] | Medline | 181 | 0.82 | 0.48 | 0.54 | 0.89 | LF | permanent | occlusal | vitro without frozen | histological | Sound (0–7); Enamel (8–14); Dentinal (> 24) |
| Kavvadia et al. 2011  [[17](#_ENREF_17)] | Embase | 111 | 0.87 | 0.38 | 0.39 | 0.87 | LF | primary | occlusal | vitro without frozen | histological | Sound ( ≤3); Enamel ( >3); Dentinal ( >40) |
| Matos et al. 2011a  [[18](#_ENREF_18)] | Medline | D1- 383 D3- 407 | 0.687 | 0.813 | 0.952 | 0.883 | LFpen | primary | occlusal | vivo | visual and operative intervention | Sound (0–4); Enamel ( >4); Dentinal ( >34) |
| Matos et al. 2011a1 [[18](#_ENREF_18)] | Medline | D1- 383 D3- 407 | 0.44 | 0.906 | 1 | 0.902 | FC | primary | occlusal | vivo | visual and operative intervention | Sound (0–1.1); Enamel (1.1-1.3), Dentinal (>1.4) |
| Neuhaus et al. 2011a  [[19](#_ENREF_19)] | Medline | 37 | 0.74 | 0.81 | 0.68 | 0.84 | LF | primary | occlusal | vitro frozen | histological | Sound (<10); Enamel (>10); Dentinal(>17) |
| Neuhaus et al. 2011a1  [[19](#_ENREF_19)] | Medline | 37 | 0.70 | 0.90 | 0.76 | 0.80 | LFpen | primary | occlusal | vitro frozen | histological | Sound (<14); Enamel (>14); Dentinal (>31) |
| Pereira et al. 2011a  [[20](#_ENREF_20)] | Scopus | 96 | 0.78 | 0.91 |  |  | LF | permanent | occlusal | vitro without frozen | histological | Enamel (>5) |
| Pereira et al. 2011a1  [[20](#_ENREF_20)] | Scopus | 96 | 0.96 | 0.38 |  |  | QLF | permanent | occlusal | vitro without frozen | histological | Enamel (Score 2 -Fluorescence loss distinctly visible without enamel broken) |
| Rando-Meirelles and de Sousa Mda L. 2011  [[21](#_ENREF_21)] | Medline | 789 | 0.64 | 0.74 |  |  | LF | permanent | occlusal | vivo | radiographic | Sound (00-20); Enamel (21-30); Dentinal (31-99) |
| Rodrigues et al. 2011a  [[22](#_ENREF_22)] | Medline | 97 | 0.70 | 0.76 | 0.63 | 0.88 | LF | permanent | occlusal | vitro frozen | histological | Sound (0–7); Enamel (8–14); Dentinal (> 24) |
| Rodrigues et al. 2011a1  [[22](#_ENREF_22)] | Medline | 97 | 0.62 | 0.76 | 0.63 | 0.87 | LFpen | permanent | occlusal | vitro frozen | histological | Sound (0–7); Enamel (8–14); Dentinal (> 24) |
| Rodrigues et al. 2011a3  [[22](#_ENREF_22)] | Medline | 97 | 0.75 | 0.7 | 0.96 | 0.7 | FC | permanent | occlusal | vitro frozen | histological | Sound (0-1.2); Enamel (1.2-1.4); Dentinal (>1.4) |
| Chu et al. 2010  [[23](#_ENREF_23)] | Medline | 144 |  |  | 0.70 | 0.84 | LF | permanent | occlusal | vivo | operative intervention | Dentinal (>40) |
| Huth, Lussi et al. 2010  [[24](#_ENREF_24)] | Medline | 117 | 0.68 | 0.7 | 0.6 | 0.84 | LFpen | permanent | approximal | vivo | visual and radiographic | Sound (<7); Enamel (>7); Dentinal (>16) |
| Novaes et al. 2010  [[25](#_ENREF_25)] | Medline | 592 | 0.295 | 0.816 | 0.516 | 0.952 | LFpen | primary | approximal | vivo | visual after tooth separation | Sound (0–5); Enamel (>5); Dentinal (>16) |
| Umemori et al. 2010  [[26](#_ENREF_26)] | Medline | 100 | 0.89 | 0.84 |  |  | LF | permanent | occlusal | vivo | operative intervention | Sound (>16,9); Enamel (>45,2); Dentinal (>57,9) |
| Abalos et al. 2009  [[27](#_ENREF_27)] | Medline | 102 |  |  | 0.89 | 0.75 | LF | permanent | occlusal | vivo | operative intervention | Dentinal (>20) |
| Apostolopoulou et al. 2009 [[28](#_ENREF_28)] | Medline | 111 | 0.9 | 0.36 | 0.36 | 0.91 | LF | primary | occlusal | vitro without frozen | histological | Enamel (>16); Dentine (>59) |
| Braga et al. 2009 [[29](#_ENREF_29)] | Medline | 131 | 0.87 | 0.25 | 0.77 | 0.71 | LFpen | primary | approximal | vitro frozen | visual e histological | Sound (0-4); Enamel (4.1-38); Dentinal (>38) |
| Diniz et al. 2009  [[30](#_ENREF_30)] | Medline | 130 |  |  | 0.7 | 0.87 | LF | permanent | occlusal | vivo | visual, radiographic and operative intervention | Sound (0–14); Enamel (15–21); Dentinal (>22) |
| Goel et al. 2009  [[31](#_ENREF_31)] | Medline | 83 | 0.81 | 1.00 | 0.77 | 0.74 | LF | primary | occlusal | vivo | histological | Sound (0-6); Enamel (7-20); Dentinal (≥35) |
| Khalife et al. 2009  [[32](#_ENREF_32)] | Medline | 60 |  |  | 0.91 | 0.38 | LF | permanent | occlusal | vivo | operative intervention | Dentinal (>30) |
| Novaes et al. 2009  [[33](#_ENREF_33)] | Medline | 621 | 0.16 | 0.96 | 0.65 | 1.00 | LFpen | primary | approximal | vivo | temporary separation (visual) | Sound (0–5); Enamel (5-16); Dentinal (>16) |
| Rodrigues et al. 2009a  [[34](#_ENREF_34)] | Medline | 148 | 0.53 | 0.92 | 0.16 | 0.89 | LF | permanent | occlusal | vitro without frozen | histological | Sound (0–7); Enamel (7.1–14); Dentinal (>24) |
| Rodrigues et al. 2009a1  [[34](#_ENREF_34)] | Medline | 179 | 0.24 | 0.92 | 0.20 | 0.94 | LF | primary | occlusal | vitro without frozen | histological | Sound (0–7); Enamel (7.1–14); Dentinal (>24) |
| Sridhar et al. 2009 [[35](#_ENREF_35)] | Medline | 50 | 1 | 0.33 | 0.87 | 0.833 | LF | permanent | occlusal | vitro without frozen | histological | Sound (<6); Enamel (6-14) |
| Barberia et al. 2008a  [[36](#_ENREF_36)] | Medline | 243 | 0.89 | 0.89 |  |  | LF | primary | occlusal | vivo | visual | Sound (0-4); Enamel (5-25); Dentinal (>26) |
| Barberia et al. 2008a1  [[36](#_ENREF_36)] | Medline | 77 | 0.40 | 0.82 |  |  | LF | permanent | occlusal | vivo | visual | Sound (0-4); Enamel (5-25); Dentinal (>26) |
| Costa et al. 2008 [[37](#_ENREF_37)] | Medline | 151 |  |  | 0.93 | 0.75 | LF | permanent | occlusal | vivo | operative intervention | Sound (0-20); Enamel (21-29); Dentinal (>30) |
| Huth et al. 2008  [[38](#_ENREF_38)] | Medline | 120 | 0.88 | 0.85 | 0.67 | 0.79 | LFpen | permanent | occlusal | vivo | operative intervention | Enamel (>12); Dentinal (>25) |
| Kavvadia and Lagouvardos 2008  [[39](#_ENREF_39)] | Medline | 405 | 0.43 | 0.88 | 0.78 | 0.63 | LF | primary | occlusal | vivo | operative intervention | Sound (0–9); Enamel (10–42); Dentinal (30–99) |
| Rocha-Cabral et al. 2008  [[40](#_ENREF_40)] | Medline | 120 | 0.88 | 0.63 | 0.77 | 0.74 | LF | primary | occlusal | vitro without frozen | histological | Sound (0 4); Enamel (5-12); Dentinal (>12) |
| Rodrigues et al. 2008a [[41](#_ENREF_41)] | Medline | 119 |  |  | 0.51 | 0.89 | LF | permanent | occlusal | vitro frozen | histological | Dentinal (>24) |
| Rodrigues et al. 2008a1  [[41](#_ENREF_41)] | Medline | 119 |  |  | 0.78 | 0.56 | LFpen | permanent | occlusal | vitro frozen | histological | Dentinal (>17) |
| Rodrigues et al. 2008a2  [[41](#_ENREF_41)] | Medline | 119 |  |  | 0.86 | 0.63 | FC | permanent | occlusal | vitro frozen | histological | Dentinal (>1.319) |
| Rodrigues et al. 2008 [[42](#_ENREF_42)] | Medline | 65 | 0.28 | 0.50 |  |  | LF | primary | occlusal | vitro without frozen | histological | Sound (0-13)0; Enamel (14-20); Dentinal (30-99) |
| Valera et al. 2008  [[43](#_ENREF_43)] | Medline | 72 |  |  | 0.33 | 1.00 | LF | permanent | occlusal | vitro without frozen | histological | Sound (0-5); Enamel (6-10); Dentinal (>21) |
| Krause et al. 2007a  [[44](#_ENREF_44)] | Medline | 94 |  |  | 0.92 | 0.53 | LF | permanent | occlusal | vivo | operative intervention | Dentinal (>36) |
| Krause et al. 2007a1  [[44](#_ENREF_44)] | Medline | 94 |  |  | 0.88 | 0.53 | LFpen | permanent | occlusal | vivo | operative intervention | Dentinal (>23) |
| Manton and Messer 2007  [[45](#_ENREF_45)] | Medline | 198 | 0.49 | 0.83 |  |  | LF | permanent | occlusal | vitro without frozen | histological | Enamel (>14) |
| Akarsu and Koprulu 2006  [[46](#_ENREF_46)] | Medline | 165 | 0.88 | 0.71 | 0.89 | 0.87 | LF | permanent | occlusal | vivo | operative intervention | Sound (0-5.5); Enamel (5.5-11.5); Dentinal (>11.5) |
| Deery et al. 2006  [[47](#_ENREF_47)] | Medline | 37 | 0.93 | 0.07 | 0.89 | 0.73 | LF | permanent | occlusal | vitro | histological | Sound (0-4.9); Enamel (5-25); Dentinal (>25.01) |
| Kuhnisch et al. 2006a1 [[48](#_ENREF_48)] | Medline | 54 |  |  | 0.80 | 0.89 | QLF | permanent | occlusal | vitro without frozen | light microscopy | Dentinal (ΔQ= -81.0 mm2*% at the QLF threshold -16% for LM) |
| Lussi and Hellwig 2006a  [[49](#_ENREF_49)] | Medline | 119 | 0.96 | 0.69 | 0.81 | 0.79 | LF | permanent | occlusal | vitro frozen | histological | (Sound (0-7); Enamel (7.1-14); Dentinal (>24) |
| Lussi and Hellwig 2006a1  [[49](#_ENREF_49)] | Medline | 119 | 0.88 | 0.77 | 0.79 | 0.84 | LFpen | permanent | occlusal | vitro frozen | histological | (Sound (0-6); Enamel (6.1-13); Dentinal (>17) |
| Lussi et al. 2006b  [[50](#_ENREF_50)] | Medline | 150 | 0.87 | 0.93 | 0.92 | 0.81 | LFpen | permanent | approximal | vitro frozen | histological | TWDG (Sound (0-9); Enamel (9.1-13); Dentinal (>22) |
| Mendes et al. 2006a  [[51](#_ENREF_51)] | Medline | 110 | 0.61 | 0.93 | 0.73 | 0.88 | LF | primary | occlusal | vitro without frozen | histological | Sound (0-7); Enamel (8-14); Dentinal (>14) |
| Olmez et al. 2006 [[52](#_ENREF_52)] | Medline | 92 | 0.93 | 1 | 0.86 | 0.80 | LF | permanent | occlusal | vivo | operative intervention | Sound (0-14); Enamel (15-20); Dentinal (21-99) |
| Reis et al. 2006a  [[53](#_ENREF_53)] | Medline | 110 | 0.71 | 0.57 | 0.78 | 0.63 | LF | permanent | occlusal | vitro without frozen | histological | Sound (0-13); Enamel (14-19); Dentinal (>20) |
| Reis et al. 2006a1 [[53](#_ENREF_53)] | Medline | 110 | 0.8 | 0.43 | 0.75 | 0.52 | LF | permanent | occlusal | vivo | histological | Sound (0-13); Enamel (14-19); Dentinal (>20) |
| Bengtson et al. 2005  [[54](#_ENREF_54)] | Medline | 87 | 0.29 | 0.98 | 0.33 | 1.00 | LF | primary | occlusal | vitro without frozen | histological | Sound (0-4); Enamel (5-12); Dentinal (>12) |
| Burin et al. 2005  [[55](#_ENREF_55)] | Medline | 105 |  |  | 0.72 | 0.67 | LF | permanent | occlusal | vitro without frozen | histological | Sound (<11); Enamel (12-16); Dentinal (>16) |
| Lussi et al. 2005a  [[56](#_ENREF_56)] | Medline | 117 |  |  | 0.98 | 0.93 | LF | permanent | occlusal | vivo | operative intervention | Sound (0-15); Enamel (16-17); Dentinal (>18) |
| Mendes et al. 2005  [[57](#_ENREF_57)] | Medline | 77 | 0.51 | 0.96 | 0.82 | 0.94 | LF | primary | smooth | vitro without frozen | histological | Sound (<3); Enamel (>3); Dentinal (>8) |
| Virajsilp et al. 2005  [[58](#_ENREF_58)] | Medline | 107 | 0.75 | 0.94 | 0.85 | 0.89 | LF | primary | approximal | vitro without frozen | histological | Sound (<2); Enamel (2); Dentinal (>4) |
| Reis et al. 2004  [[59](#_ENREF_59)] | Medline | 45 |  |  | 0.46 | 0.9 | LF | permanent | occlusal | vitro without frozen | histological | Sound (0–4); Enamel (5–20); Dentinal (>20) |
| Anttonen et al. 2003  [[60](#_ENREF_60)] | Medline | 613 |  |  | 0.92 | 0.82 | LF | permanent | occlusal | vivo | visual and operative intervention | Dentinal (>30) |
| Baseren and Gokalp 2003  [[61](#_ENREF_61)] | Medline | 31 | 1 | 0.74 | 1 | 0.92 | LF | permanent | occlusal | vitro without frozen | histological | Enamel (14–19); Dentinal (> 20) |
| Chong et al. 2003 [[62](#_ENREF_62)] | Medline | 320 | 0.89 | 0.56 | 0.82 | 0.36 | LF | permanent | occlusal | vitro without frozen | visual and radiographic | Sound (<5); Enamel (5-25); Dentinal (>26) |
| Cortes et al. 2003 [[63](#_ENREF_63)] | Medline | 152 | 0.73 | 0.85 | 0.84 | 0.67 | LF | permanent | occlusal | vitro without frozen | histological | Sound (<17); Enamel (>17); Dentinal(>23) |
| Francescut and Lussi 2003a  [[64](#_ENREF_64)] | Medline | 95 | 0.75 | 0.68 | 0.82 | 0.85 | LF | primary | occlusal | vitro without frozen | histological | Enamel (≥5); Dentinal (≥13) |
| Francescut and Lussi 2003a1  [[64](#_ENREF_64)] | Medline | 95 | 0.77 | 0.49 | 0.73 | 0.65 | LF | permanent | occlusal | vitro without frozen | histological | Enamel (≥6); Dentinal (≥10) |
| Heinrich-Weltzien et al. 2003 [[65](#_ENREF_65)] | Medline | 248 |  |  | 0.95 | 0.58 | LF | permanent | occlusal | vivo | operative intervention | Dentinal (>18) |
| Rocha et al. 2003  [[66](#_ENREF_66)] | Medline | 50 | 0.6 | 0.90 | 0.73 | 0.95 | LF | primary | occlusal | vivo | histological | Sound (0–5); Enamel (6–14); Dentinal (21–99) |
| Bamzahim et al. 2002  [[67](#_ENREF_67)] | Medline | 87 |  |  | 0.8 | 1.00 | LF | permanent | occlusal | vitro without frozen | histological | Dentinal (>18) |
| Ouellet et al. 2002  [[68](#_ENREF_68)] | Medline | 100 |  |  | 0.73 | 0.55 | LF | permanent | occlusal | vitro without frozen | clinical exam e photograph with caries-detecting dye | Dentinal (>16) |
| Attrill and Ashley 2001  [[69](#_ENREF_69)] | Medline | 58 |  |  | 0.77 | 0.82 | LF | primary | occlusal | vitro without frozen | histological | Sound (0–9); Enamel (10–17); Dentinal (18–99) |
| Lussi et al. 2001  [[70](#_ENREF_70)] | Medline | 332 |  |  | 0.92 | 0.86 | LF | permanent | occlusal | vivo | operative intervention | Enamel (>14); Dentinal (>20) |
| (Pereira et al. 2001  [[71](#_ENREF_71)] | Medline | 230 |  |  | 0.2 | 0.98 | LF | permanent | occlusal | vitro without frozen | histological | Dentinal (10-11) |
| Sheehy et al. 2001  [[72](#_ENREF_72)] | Medline | 170 | 0.70 | 0.87 | 0.94 | 0.90 | LF | permanent | occlusal | vivo | visual | Sound (0-14) Enamel (15-20); Dentinal (>20) |
| Shi et al. 2001a [[73](#_ENREF_73)] | Medline | 71 |  |  | 0.75 | 0.96 | LF | permanent | smooth | vitro without frozen | histological | Dentinal (>9) |
| Shi et al. 2001a1 [[73](#_ENREF_73)] | Medline | 71 |  |  | 0.83 | 0.98 | QLF | permanent | smooth | vitro without frozen | histological | Dentinal (>25%) |
| Shi et al. 2000  [[74](#_ENREF_74)] | Medline | 70 | 0.46 | 0.95 | 0.82 | 1 | LF | permanent | occlusal | vitro without frozen | histological | Enamel (6.8-7.1); Dentinal (21.5- 22.1) |
| (Lussi et al. 1999 [[75](#_ENREF_75)] | Medline | 105 | 0.83 | 0.72 | 0.84 | 0.79 | LF | permanent | occlusal | vitro without frozen | histological | Sound (0-4); Enamel (4.01-10); Dentinal (>10.01) |
|  |  |  |  |  |  |  |  |  |  |  |  |  |

1. Abalos C, Mendoza A, Jimenez-Planas A, Guerrero E, Chaparro A, et al. (2012) Performance of laser fluorescence for the detection of enamel caries in non-cavitated occlusal surfaces: clinical study with total validation of the sample. Am J Dent 25: 44-48.

2. Achilleos EE, Rahiotis C, Kakaboura A, Vougiouklakis G (2012) Evaluation of a new fluorescence-based device in the detection of incipient occlusal caries lesions. Lasers Med Sci.

3. Chen J, Qin M, Ma W, Ge L (2012) A clinical study of a laser fluorescence device for the detection of approximal caries in primary molars. Int J Paediatr Dent 22: 132-138.

4. Diniz MB, Boldieri T, Rodrigues JA, Santos-Pinto L, Lussi A, et al. (2012) The performance of conventional and fluorescence-based methods for occlusal caries detection: an in vivo study with histologic validation. J Am Dent Assoc 143: 339-350.

5. Jablonski-Momeni A, Rosen SM, Schipper HM, Stoll R, Roggendorf MJ, et al. (2012) Impact of measuring multiple or single occlusal lesions on estimates of diagnostic accuracy using fluorescence methods. Lasers Med Sci 27: 343-352.

6. Jablonski-Momeni A, Liebegall F, Stoll R, Heinzel-Gutenbrunner M, Pieper K (2012) Performance of a new fluorescence camera for detection of occlusal caries in vitro. Lasers Med Sci.

7. Matos R, Novaes TF, Reyes A, De Benedetto MS, Mendes FM, et al. (2012) Influence of cross-infection control methods on performance of pen-type laser fluorescence in detecting occlusal caries lesions in primary teeth. Lasers Med Sci.

8. Novaes TF, Matos R, Gimenez T, Braga MM, MS DEB, et al. (2012) Performance of fluorescence-based and conventional methods of occlusal caries detection in primary molars - an in vitro study. Int J Paediatr Dent.

9. Rechmann P, Charland D, Rechmann BM, Featherstone JD (2012) Performance of laser fluorescence devices and visual examination for the detection of occlusal caries in permanent molars. J Biomed Opt 17: 036006.

10. Seremidi K, Lagouvardos P, Kavvadia K (2012) Comparative in vitro validation of VistaProof and DIAGNOdent pen for occlusal caries detection in permanent teeth. Oper Dent 37: 234-245.

11. Aktan AM, Cebe MA, Ciftci ME, Sirin Karaarslan E (2011) A novel LED-based device for occlusal caries detection. Lasers Med Sci.

12. Bittar DG, Gimenez T, Morais CC, De Benedetto MS, Braga MM, et al. (2011) Influence of moisture and plaque on the performance of a laser fluorescence device in detecting caries lesions in primary teeth. Lasers in Medical Science: 1-6.

13. de Paula AB, Campos JA, Diniz MB, Hebling J, Rodrigues JA (2011) In situ and in vitro comparison of laser fluorescence with visual inspection in detecting occlusal caries lesions. Lasers Med Sci 26: 1-5.

14. Diniz MB, Sciasci P, Rodrigues JA, Lussi A, Cordeiro RCL (2011) Influence of different professional prophylactic methods on fluorescence measurements for detection of occlusal caries. Caries Research 45: 264-268.

15. Jablonski-Momeni A, Schipper HM, Rosen SM, Heinzel-Gutenbrunner M, Roggendorf MJ, et al. (2011) Performance of a fluorescence camera for detection of occlusal caries in vitro. Odontology 99: 55-61.

16. Jablonski-Momeni A, Ricketts DN, Rolfsen S, Stoll R, Heinzel-Gutenbrunner M, et al. (2011) Performance of laser fluorescence at tooth surface and histological section. Lasers Med Sci 26: 171-178.

17. Kavvadia K, Lagouvardos P, Apostolopoulou D (2011) Combined validity of DIAGNOdent™ and visual examination for in vitro detection of occlusal caries in primary molars. Lasers in Medical Science: 1-7.

18. Matos R, Novaes TF, Braga MM, Siqueira WL, Duarte DA, et al. (2011) Clinical performance of two fluorescence-based methods in detecting occlusal caries lesions in primary teeth. Caries Res 45: 294-302.

19. Neuhaus KW, Rodrigues JA, Hug I, Stich H, Lussi A (2011) Performance of laser fluorescence devices, visual and radiographic examination for the detection of occlusal caries in primary molars. Clinical Oral Investigations 15: 635-641.

20. Pereira AC, Eggertsson H, González-Cabezas C, Zero DT, Eckert GJ, et al. (2011) Quantitative light-induced fluorescence (QLF) in relation to other technologies and conventional methods for detecting occlusal caries in permanent teeth. Brazilian Journal of Oral Sciences 10: 27-32.

21. Rando-Meirelles MP, de Sousa Mda L (2011) Using laser fluorescence (DIAGNOdent) in surveys for the detection of noncavitated occlusal dentine caries. Community Dent Health 28: 17-21.

22. Rodrigues JA, Hug I, Neuhaus KW, Lussi A (2011) Light-emitting diode and laser fluorescence-based devices in detecting occlusal caries. J Biomed Opt 16: 107003.

23. Chu CH, Lo EC, You DS (2010) Clinical diagnosis of fissure caries with conventional and laser-induced fluorescence techniques. Lasers Med Sci 25: 355-362.

24. Huth KC, Lussi A, Gygax M, Thum M, Crispin A, et al. (2010) In vivo performance of a laser fluorescence device for the approximal detection of caries in permanent molars. J Dent 38: 1019-1026.

25. Novaes TF, Matos R, Raggio DP, Imparato JC, Braga MM, et al. (2010) Influence of the Discomfort Reported by Children on the Performance of Approximal Caries Detection Methods. Caries Res 44: 465-471.

26. Umemori S, Tonami K, Nitta H, Mataki S, Araki K (2010) The possibility of digital imaging in the diagnosis of occlusal caries. Int J Dent 2010: 860515.

27. Abalos C, Herrera M, Jimenez-Planas A, Llamas R (2009) Performance of laser fluorescence for detection of occlusal dentinal caries lesions in permanent molars: an in vivo study with total validation of the sample. Caries Res 43: 137-141.

28. Apostolopoulou D, Lagouvardos P, Kavvadia K, Papagiannoulis L (2009) Histological validation of a laser fluorescence device for occlusal caries detection in primary molars. Eur Arch Paediatr Dent 10 Suppl 1: 11-15.

29. Braga MM, Morais CC, Nakama RC, Leamari VM, Siqueira WL, et al. (2009) In vitro performance of methods of approximal caries detection in primary molars. Oral Surg Oral Med Oral Pathol Oral Radiol Endod 108: e35-41.

30. Diniz MB, Rodrigues JA, de Paula AB, Cordeiro Rde C (2009) In vivo evaluation of laser fluorescence performance using different cut-off limits for occlusal caries detection. Lasers Med Sci 24: 295-300.

31. Goel A, Chawla HS, Gauba K, Goyal A (2009) Comparison of validity of DIAGNOdent with conventional methods for detection of occlusal caries in primary molars using the histological gold standard: an in vivo study. J Indian Soc Pedod Prev Dent 27: 227-234.

32. Khalife MA, Boynton JR, Dennison JB, Yaman P, Hamilton JC (2009) In vivo evaluation of DIAGNOdent for the quantification of occlusal dental caries. Oper Dent 34: 136-141.

33. Novaes TF, Matos R, Braga MM, Imparato JC, Raggio DP, et al. (2009) Performance of a pen-type laser fluorescence device and conventional methods in detecting approximal caries lesions in primary teeth--in vivo study. Caries Res 43: 36-42.

34. Rodrigues JA, Diniz MB, Josgrilberg EB, Cordeiro RC (2009) In vitro comparison of laser fluorescence performance with visual examination for detection of occlusal caries in permanent and primary molars. Lasers Med Sci 24: 501-506.

35. Sridhar N, Tandon S, Rao N (2009) A comparative evaluation of DIAGNOdent with visual and radiography for detection of occlusal caries: an in vitro study. Indian J Dent Res 20: 326-331.

36. Barberia E, Maroto M, Arenas M, Silva CC (2008) A clinical study of caries diagnosis with a laser fluorescence system. J Am Dent Assoc 139: 572-579.

37. Costa AM, De Paula LM, Bezerra ACB (2008) Use of diagnodent® for diagnosis of non-cavitated occlusal dentin caries. Journal of Applied Oral Science 16: 18-23.

38. Huth KC, Neuhaus KW, Gygax M, Bucher K, Crispin A, et al. (2008) Clinical performance of a new laser fluorescence device for detection of occlusal caries lesions in permanent molars. J Dent 36: 1033-1040.

39. Kavvadia K, Lagouvardos P (2008) Clinical performance of a diode laser fluorescence device for the detection of occlusal caries in primary teeth. Int J Paediatr Dent 18: 197-204.

40. Rocha-Cabral RM, Mendes FM, Miura F, Ribeiro Ada C, Braga MM, et al. (2008) Autoclaving and battery capacity influence on laser fluorescence measurements. Acta Odontol Scand 66: 122-127.

41. Rodrigues JA, Hug I, Diniz MB, Lussi A (2008) Performance of fluorescence methods, radiographic examination and ICDAS II on occlusal surfaces in vitro. Caries Res 42: 297-304.

42. Rodrigues JA, Hug I, Diniz MB, Lussi A (2008) Performance of fluorescence methods, radiographic examination and ICDAS II on occlusal surfaces in vitro. Caries Research 42: 297-304.

43. Valera FB, Pessan JP, Valera RC, Mondelli J, Percinoto C (2008) Comparison of visual inspection, radiographic examination, laser fluorescence and their combinations on treatment decisions for occlusal surfaces. Am J Dent 21: 25-29.

44. Krause F, Jepsen S, Braun A (2007) Comparison of two laser fluorescence devices for the detection of occlusal caries in vivo. Eur J Oral Sci 115: 252-256.

45. Manton DJ, Messer LB (2007) The effect of pit and fissure sealants on the detection of occlusal caries in vitro. Eur Arch Paediatr Dent 8: 43-48.

46. Akarsu S, Koprulu H (2006) In vivo comparison of the efficacy of DIAGNOdent by visual inspection and radiographic diagnostic techniques in the diagnosis of occlusal caries. J Clin Dent 17: 53-58.

47. Deery C, Iloya J, Nugent ZJ, Srinivasan V (2006) Effect of placing a clear sealant on the validity and reproducibility of occlusal caries detection by a laser fluorescence device: an in vitro study. Caries Res 40: 186-193.

48. Kuhnisch J, Ifland S, Tranaeus S, Angmar-Mansson B, Hickel R, et al. (2006) Establishing quantitative light-induced fluorescence cut-offs for the detection of occlusal dentine lesions. Eur J Oral Sci 114: 483-488.

49. Lussi A, Hellwig E (2006) Performance of a new laser fluorescence device for the detection of occlusal caries in vitro. Journal of Dentistry 34: 467-471.

50. Lussi A, Hack A, Hug I, Heckenberger H, Megert B, et al. (2006) Detection of approximal caries with a new laser fluorescence device. Caries Res 40: 97-103.

51. Mendes FM, Ganzerla E, Nunes AF, Puig AV, Imparato JC (2006) Use of high-powered magnification to detect occlusal caries in primary teeth. Am J Dent 19: 19-22.

52. Olmez A, Tuna D, Oznurhan F (2006) Clinical evaluation of diagnodent in detection of occlusal caries in children. J Clin Pediatr Dent 30: 287-291.

53. Reis A, Mendes FM, Angnes V, Angnes G, Grande RH, et al. (2006) Performance of methods of occlusal caries detection in permanent teeth under clinical and laboratory conditions. J Dent 34: 89-96.

54. Bengtson AL, Gomes AC, Mendes FM, Cichello LR, Bengtson NG, et al. (2005) Influence of examiner's clinical experience in detecting occlusal caries lesions in primary teeth. Pediatr Dent 27: 238-243.

55. Burin C, Loguercio AD, Grande RH, Reis A (2005) Occlusal caries detection: a comparison of a laser fluorescence system and conventional methods. Pediatr Dent 27: 307-312.

56. Lussi A, Longbottom C, Gygax M, Braig F (2005) Influence of professional cleaning and drying of occlusal surfaces on laser fluorescence in vivo. Caries Res 39: 284-286.

57. Mendes FM, Siqueira WL, Mazzitelli JF, Pinheiro SL, Bengtson AL (2005) Performance of DIAGNOdent for detection and quantification of smooth-surface caries in primary teeth. J Dent 33: 79-84.

58. Virajsilp V, Thearmontree A, Aryatawong S, Paiboonwarachat D (2005) Comparison of proximal caries detection in primary teeth between laser fluorescence and bitewing radiography. Pediatr Dent 27: 493-499.

59. Reis A, Zach VL, Jr., de Lima AC, de Lima Navarro MF, Grande RH (2004) Occlusal caries detection: a comparison of DIAGNOdent and two conventional diagnostic methods. J Clin Dent 15: 76-82.

60. Anttonen V, Seppa L, Hausen H (2003) Clinical study of the use of the laser fluorescence device DIAGNOdent for detection of occlusal caries in children. Caries Res 37: 17-23.

61. Baseren NM, Gokalp S (2003) Validity of a laser fluorescence system (DIAGNOdent) for detection of occlusal caries in third molars: an in vitro study. J Oral Rehabil 30: 1190-1194.

62. Chong MJ, Seow WK, Purdie DM, Cheng E, Wan V (2003) Visual-tactile examination compared with conventional radiography, digital radiography, and Diagnodent in the diagnosis of occlusal occult caries in extracted premolars. Pediatr Dent 25: 341-349.

63. Cortes DF, Ellwood RP, Ekstrand KR (2003) An in vitro comparison of a combined FOTI/visual examination of occlusal caries with other caries diagnostic methods and the effect of stain on their diagnostic performance. Caries Res 37: 8-16.

64. Francescut P, Lussi A (2003) Correlation between fissure discoloration, Diagnodent measurements, and caries depth: an in vitro study. Pediatr Dent 25: 559-564.

65. Heinrich-Weltzien R, Kühnisch J, Oehme T, Ziehe A, Stösser L, et al. (2003) Comparison of Different DIAGNOdent Cut-off Limits for In Vivo Detection of Occlusal Caries. Operative Dentistry 28: 672-680.

66. Rocha RO, Ardenghi TM, Oliveira LB, Rodrigues CR, Ciamponi AL (2003) In vivo effectiveness of laser fluorescence compared to visual inspection and radiography for the detection of occlusal caries in primary teeth. Caries Res 37: 437-441.

67. Bamzahim M, Shi XQ, Angmar-Mansson B (2002) Occlusal caries detection and quantification by DIAGNOdent and Electronic Caries Monitor: in vitro comparison. Acta Odontol Scand 60: 360-364.

68. Ouellet A, Hondrum SO, Pietz DM (2002) Detection of occlusal carious lesions. Gen Dent 50: 346-350.

69. Attrill DC, Ashley PF (2001) Occlusal caries detection in primary teeth: a comparison of DIAGNOdent with conventional methods. Br Dent J 190: 440-443.

70. Lussi A, Megert B, Longbottom C, Reich E, Francescut P (2001) Clinical performance of a laser fluorescence device for detection of occlusal caries lesions. European Journal of Oral Sciences 109: 14-19.

71. Pereira AC, Verdonschot EH, Huysmans MC (2001) Caries detection methods: can they aid decision making for invasive sealant treatment? Caries Res 35: 83-89.

72. Sheehy EC, Brailsford SR, Kidd EA, Beighton D, Zoitopoulos L (2001) Comparison between visual examination and a laser fluorescence system for in vivo diagnosis of occlusal caries. Caries Res 35: 421-426.

73. Shi XQ, Tranaeus S, Angmar-Mansson B (2001) Comparison of QLF and DIAGNOdent for quantification of smooth surface caries. Caries Res 35: 21-26.

74. Shi XQ, Welander U, Angmar-Mansson B (2000) Occlusal caries detection with KaVo DIAGNOdent and radiography: an in vitro comparison. Caries Res 34: 151-158.

75. Lussi A, Imwinkelried S, Pitts N, Longbottom C, Reich E (1999) Performance and reproducibility of a laser fluorescence system for detection of occlusal caries in vitro. Caries Res 33: 261-266.
